# Overexpression of a Plasma Membrane-Localized *Sb*SRP-Like Protein Enhances Salinity and Osmotic Stress Tolerance in Transgenic Tobacco

**DOI:** 10.3389/fpls.2017.00582

**Published:** 2017-04-20

**Authors:** Pushpika Udawat, Rajesh K. Jha, Avinash Mishra, Bhavanath Jha

**Affiliations:** ^1^Marine Biotechnology and Ecology Division, Council of Scientific and Industrial Research-Central Salt and Marine Chemicals Research InstituteBhavnagar, India; ^2^Academy of Scientific and Innovative Research, Council of Scientific and Industrial ResearchNew Delhi, India

**Keywords:** abiotic stress tolerance, drought, halophytes, novel gene, salinity, transgenic

## Abstract

An obligate halophyte, *Salicornia brachiata* grows in salt marshes and is considered to be a potential resource of salt- and drought-responsive genes. It is important to develop an understanding of the mechanisms behind enhanced salt tolerance. To increase this understanding, a novel *SbSRP* gene was cloned, characterized, over-expressed, and functionally validated in the model plant *Nicotiana tabacum*. The genome of the halophyte *S. brachiata* contains two homologs of an intronless *SbSRP* gene of 1,262 bp in length that encodes for a stress-related protein. An *in vivo* localization study confirmed that *Sb*SRP is localized on the plasma membrane. Transgenic tobacco plants (T1) that constitutively over-express the *SbSRP* gene showed improved salinity and osmotic stress tolerance. In comparison to Wild Type (WT) and Vector Control (VC) plants, transgenic lines showed elevated relative water and chlorophyll content, lower malondialdehyde content, lower electrolyte leakage and higher accumulation of proline, free amino acids, sugars, polyphenols, and starch under abiotic stress treatments. Furthermore, a lower build-up of H_2_O_2_ content and superoxide-radicals was found in transgenic lines compared to WT and VC plants under stress conditions. Transcript expression of *Nt-APX* (ascorbate peroxidase), *Nt-CAT* (catalase), *Nt-SOD* (superoxide dismutase), *Nt-DREB* (dehydration responsive element binding factor), and *Nt-AP2* (apetala2) genes was higher in transgenic lines under stress compared to WT and VC plants. The results suggested that overexpression of membrane-localized *Sb*SRP mitigates salt and osmotic stress in the transgenic tobacco plant. It was hypothesized that *Sb*SRP can be a transporter protein to transmit the environmental stimuli downward through the plasma membrane. However, a detailed study is required to ascertain its exact role in the abiotic stress tolerance mechanism. Overall, *SbSRP* is a potential candidate to be used for engineering salt and osmotic tolerance in crops.

## Introduction

About 450 million small-scale producers around the world are dependent on agriculture for their sustenance. The Food and Agriculture Organization of the United Nations (FAO, 2016) estimates that feeding the world's population will require a 60% expansion in overall agriculture production by 2050. Many resources are needed for sustainable food security as the challenges faced by industries are huge and there is an urgent need for crop improvements to mitigate crop failure during unfavorable conditions (FAO, 2016). Plants encounter a wide range of environmental stresses during their lifecycles and they have evolved different mechanisms to combat these stresses, for example through modulating their physiology and interactive molecular and cellular alterations (Knight and Knight, 2001). The Intergovernmental Panel on Climate Change (IPCC) reported that high temperatures, salinity, floods, drought, and deterioration of arable land severely affect the agricultural economy in the developing world (Annan et al., 2009).

According to a report, about 800 million ha of land are salt-affected worldwide and soil salinity is gradually increasing (Munns, 2005; Tuteja et al., 2011). Furthermore, it has also been predicted that drought stress will become more frequent because of the long-term effects of global warming. The impact of abiotic stresses differs regionally and depends on the adopted agricultural practices. An adverse impact is envisaged for the agricultural economy of developing countries, therefore, major precautionary steps are needed to develop adaptive strategies for sustainable agriculture according to the changing environment (Ashraf and Foolad, 2007; Tuteja et al., 2011). The majority of crops are glycophytes, which means they are not able to grow in soil that has a high salt concentration (Shabala, 2013). The extent to which plants can endure high salinity varies among species, and a way to differentiate these species is to establish whether they profit from a large amount (100 mM or more) of NaCl in the soil (Shabala and Mackay, 2011). Salt tolerance is usually measured as the percentage biomass gain by plants in the saline environment compared to control conditions over a definite time period (Munns, 2002). Most commonly, the ions that cause high salinity are Na^+^ and Cl^−^ as they prevail in seawater. Halophytes utilize these ions for a large portion of their osmotic change (Flowers et al., 2010). Plants have evolutionarily conserved mechanisms to endure harsh conditions by expressing enzymes, transcription regulators and other factors that function in pathways directed by phytohormones, such as abscisic acid (ABA), and second messengers, such as Ca^2+^ (Mukhopadhyay and Tyagi, 2015).

The complete waterfront zone of Gujarat (India) is gradually becoming increasingly saline due to salt farming, poor agricultural practices, urbanization and poor environmental practices (Jha et al., 2011). *Salicornia brachiata* is an obligate halophyte that belongs to the Amaranthaceae family. It is a leafless annual succulent plant and is abundant in salt marshes on the Gujarat coast of India. *S. brachiata* has the ability to grow in a wide range of NaCl concentrations (0.1–2.0 M) and also requires NaCl for *in vitro* regeneration (Joshi et al., 2012). This unusual characteristics, alongside other factors such as its oligosaccharides, proteome and metabolites, provides an opportunity to investigate its salt tolerance system (Jha et al., 2012; Joshi et al., 2012; Mishra et al., 2013, 2015; Patel et al., 2016a). Different salt tolerance mechanisms have been reported from halophytes (Jha et al., 2011; Chaturvedi et al., 2014; Singh et al., 2014a; Udawat et al., 2016) and several EST databases have been created for numerous halophytes, including *S. brachiata* (Jha et al., 2009), *Alfalfa* (Jin et al., 2010), and *Chenopodium album* (Gu et al., 2011).

Molecular processes that control Na^+^ compartmentalization in vacuoles receive much attention, while other key procedures in the tissue tolerance of Na^+^ and Cl^−^ and osmotic change are neglected (Munns and Tester, 2008). From a developmental perspective, all resistance systems are modified and genotype specific (Vinocur and Altman, 2005). Several candidate genes and promoters responsible for enhanced abiotic stress tolerance have been cloned from *Salicornia* and characterized in model organisms and crop plants such as jatropha, castor, cumin, and groundnut (Chaturvedi et al., 2012; Joshi et al., 2013; Pandey et al., 2013, 2016; Singh et al., 2014b; Tiwari et al., 2014, 2015a,b, 2016; Udawat et al., 2014; Patel et al., 2015). However, it is challenging to identify key genes in the stress tolerance mechanism. A number of novel genes have been characterized, such as *SbUSP, SbSDR1, MsZEP*, and *IbZFP1*, but other novel or uncharacterized genes may play a critical role in stress tolerance (Singh et al., 2016; Udawat et al., 2016; Wang et al., 2016; Zhang et al., 2016). The EST database contains about 30% novel or uncharacterized clone sequences for *Salicornia*. The gene clone Sal-C-64 (EB484712) exhibited high expression under salt stress but shows no significant homology with the existing database. It was speculated that this gene might be involved in the abiotic stress tolerance mechanism; therefore, it was selected for functional validation. The full-length gene encodes for a protein that is designated as S*alicornia* b*rachiata* stress-related protein (*Sb*SRP).

Despite these developments, numerous difficulties remain, not just in explaining the stress response mechanism in plants, but also in utilization genes from halophytes to increase the salt tolerance of crop plants (Qin et al., 2011). In order to better understand the mechanism of salt tolerance, the role of a number of genes involved in plant homeostasis was investigated. These genes modulate physiology by compartmentalizing ions for osmotic alteration, combining perfect solutes, gathering fundamental supplements (especially K^+^) when under high salinity conditions (increased concentration of Na^+^ ions), confining the passage of saline ions into the transpiration stream, and managing transpiration under saline condition (Flowers et al., 2010). To accomplish the genetic engineering of plants with improved abiotic stress tolerance, a continuous effort to find “potential genes” is crucial in order to add to stress endurance in transgenic plants (Lee et al., 2012). The current study will identify novel salt determinants or master switches involved in ion homeostasis and osmobalance of halophytic plants. A deeper understanding of the underlying mechanisms of enhanced salt tolerance is important for the development of transgenic plants. Therefore, to understand the mechanisms responsible for improved abiotic stress tolerance of *S. brachiata*, a novel *SbSRP* gene was cloned, characterized, over-expressed and functionally validated in the model plant *N. tabacum*.

## Materials and methods

### Cloning and *in-silico* analysis

A novel EST clone (Sal-C-64; EB484712) was used to design primers (Table S1). The gene was converted to full length using RACE (rapid amplification of cDNA ends) and was sequenced and analyzed. The NCBI (National Center for Biotechnology Information) database was used to search for nucleotide and protein homologs. The secondary structure was predicted using ExPASy tools. Amino acid sequences deduced from nucleotide sequences were imported to the ProtParam tool of the ExPASy server (Artimo et al., 2012) for primary analysis and the PSIPRED server (Buchan et al., 2010) for the prediction and analysis of the secondary structure. The functional activity of *Sb*SRP was predicted using *TrSSP* (Mishra et al., 2014). The amino acid sequences were subjected to BLAST (Basic Local Alignment Search Tool) and compared with the Protein Data Bank (PDB) and Conserved Domain Data bank (CDD). The phylogram study was accomplished using the Maximum Likelihood (ML) statistical method based on the JTT matrix-based model using Molecular Evolutionary Genetics Analyses version 6 [MEGA6] (Tamura et al., 2013).

### Transcript profiling

For transcript profiling, 1-month-old seedlings were transferred to a hydroponic culture medium containing ¼ strength MS basal medium and grown *in vitro* with 8/16 h dark/light cycle at 25°C for 15 days. Different NaCl (0.05, 0.10, 0.25, 0.50, and 1.00 M) stress treatments were given to plants for 24 h by transferring plants grown under equivalent condition to new hydroponic culture media (¼ MS containing different NaCl concentrations). In the second set of experiments, different abiotic stresses (salt, 250 mM; desiccation; heat, 45°C; cold, 4°C) were applied for different time periods (2, 6, 12, 24 h) to plants. Total RNA was isolated from control and stressed plant samples using the GITC method (Chomczynski and Sacchi, 1987) and quantified with a Nanodrop spectrophotometer (NanoDrop, USA). cDNA was prepared using a ImpromII reverse transcriptase first strand cDNA synthesis kit (Promega, USA). The quantitative real-time PCR (qRT-PCR) reactions were carried out with gene-specific primers and β-tubulin was used as the internal reference gene (Table S1). At the end of the PCR cycles, products were retained through a melt curve analysis to check the specificity of PCR amplification in qRT-PCR. The amplified products were checked on 1% agarose gel to confirm the expected size. The qRT-PCR data were analyzed using the comparative C_T_ method, and the relative fold-gene expression (2^−ΔΔC_T_^) of the *SbSRP* gene in stressed plants in comparison to control plants (without treatment) was determined after normalizing with internal control β-tubulin C_T_-values (Livak and Schmittgen, 2001; Schmittgen and Livak, 2008).

### Subcellular localization

A translational fusion cassette of *SbSRP* along with RFP (red fluorescent protein) was made to study subcellular localization. The *SbSRP* open reading frame (ORF) was amplified, cloned into pENTER/D-TOPO (Invitrogen, USA) and sequenced (Table S1). An LR recombination reaction was performed between an attL-containing pENTER/D-TOPO-*SbSRP* recombinant vector and an attR-containing pSITE-4CA (RFP) destination vector using LR Clonase II enzyme mix (Invitrogen, USA). Positive clones were selected, confirmed by PCR and transferred to onion epidermal cells using a gene gun (PDS-1000/He Biolistic, Biorad, USA). Vector pSITE-4CA:RFP was also transformed and used as a control. Transformants were incubated in the dark for 12 h and the transient expression of RFP was observed using an epifluorescence microscope (Axio Imager, Carl Zeiss AG, Germany).

### Genomic organization

The *SbSRP* was amplified and the gene copy number was determined using DNA gel blot analysis (Joshi et al., 2011; Table S1). Genomic DNA of *Salicornia* was extracted using a modified CTAB (N-cetyl-N, N, Ntrimethyl ammonium bromide) method (Bubner et al., 2004) and was quantified (ND-1000, Nanodrop Technology, USA) and digested with different restriction enzymes (*EcoR*I, *Sac*I, *Sma*I, and *Hind*III). Digested DNA was transferred to a Hybond N^+^ membrane (Amersham Pharmacia, UK) using an alkaline transfer buffer (0.4 N NaOH along with 1 M NaCl). The blot was hybridized with PCR-generated DIG-11-dUTP labeled *SbSRP* probe overnight at 42°C (Table S1). Signals were obtained using CDP-Star as a chemiluminescent substrate and detected on X-ray film.

### Genetic transformation of tobacco plants and molecular confirmation

The *SbSRP* ORF was amplified (Table S1) and cloned in pCAMBIA1301 under the control of a 35S promoter *via* an intermediate pRT101 plant expression vector and the recombinant pCAMBIA1301:35S:*Sb*SRP vector was mobilized into *Agrobacterium tumefaciens* (strain LBA4404). Tobacco (*Nicotiana tabacum* cv. Petit Havana) plants were transformed by the leaf disc method (Horsch et al., 1985) and transgenic plants (T0) were obtained using standard tissue culture methods. The T1 transgenic lines, obtained from T0 plants, were subjected to PCR and southern analysis for the confirmation of trans-gene (*SbSRP*) integration.

### Semi-quantitative RT-PCR

The total RNA was extracted using the GITC method from six transgenic lines (L2, L7, L10, L16, and L17), vector control (VC) and wild-type (WT) plants (abiotic stressed and unstressed) and quantified (ND-1000). The mRNA was converted to cDNA using Superscript II RT (Invitrogen, USA). The β-tubulin gene was used as an internal reference gene and resulting cDNA of both (*SbSRP* and β-tubulin) were amplified using PCR (Table S1). The semi-quantitative RT-PCR was performed three times independently and amplicons were observed through agarose gel electrophoresis. Three transgenic lines (L2, L7, and L16) showed better over-expression of the *SbSRP* gene and so were selected for further analysis.

### Analyses of transgenic plants

The copy number of the *SbSRP* gene in transgenic lines was determined by DNA gel blotting (Joshi et al., 2011) as described above using gene-specific probes (Table S1). The T1 seeds of *SbSRP* over-expressing transgenic lines (L2, L7, and L16) along with VC and WT plants were germinated on MS media that contained NaCl (200 mM) or Polyethylene glycol (10% PEG) under controlled conditions. The seedling growth percentage was calculated using the following equation:-

$$\begin{array}{l} Percent\;seedling\;growth=(Number\;of\;seeds\;grown/\\ \;Total\;number\;of\;seeds)\times 100 \end{array}$$

T1 seedlings that were uniform in size and germinated on MSB containing hygromycin (20 mg L^−1^) were transferred to a hydroponic system with ½ strength MS media, incubated for 45 days, and then subjected to stress treatment (for 24 h under 200 mM NaCl or 10% PEG) for biochemical and physiological analysis.

### Quantification of osmotic adjustment

Leaf segments of 45 day old treated and untreated plants were collected and incubated overnight at −20°C. The next day, plant samples were thawed and centrifuged at 13,000 rpm for 10 min at 4°C. The cell sap extract was used to determine the cellular osmotic potential of the sample using a vapor pressure osmometer (VAPRO, Wescor Inc., USA).

### Relative water content (RWC)

Leaf fresh weight was measured from treated and untreated, control (WT and VC), and transgenic plants, following overnight incubation of leaf samples in deionized water. The next day, turgid weight, and dry weight were determined after drying at a constant temperature (40°C). Relative water content (RWC, %) was calculated using the following equation:

$$\begin{array}{l} RWC\;(\%)=100\times [(Fresh\;weight-Dry\;weight)/\\ \;(Turgid\;weight-Dry\;weight)] \end{array}$$

### Leaf senescence assay and quantification of chlorophyll content

Similar sized leaf discs (*n* = 10) from 45 day old control and transgenic plants were floated in ½ strength MS (control) media supplemented with NaCl (100, 150, or 200 mM) or PEG (10 or 20%) and incubated for 8 days under controlled conditions. The effects of salt and osmotic stress were examined by visually observing phenotypic alteration amongst different leaf segments. In addition, total chlorophyll content was calculated for leaf discs per gram of tissue fresh weight (Inskeep and Bloom, 1985).

### XTT and TTC assay

Leaf segments (of a uniform size and weight) of 45 day old plants (treated and untreated, control and transgenic) were used to quantify the accumulation of superoxide radicals (after stress) using a 2,3-bis-(2-methoxy-4-nitro-5-sulfophenyl)-2H-tetrazolium-5-carboxanilide (XTT) assay (Hema et al., 2014). Leaf segments were kept in a potassium phosphate buffer (20 mM, pH 6.0) containing 500 μM XTT for 5 h and the increase in absorbance was measured at 470 nm. Likewise, leaf segments (of equal weight and size) of 45 day old treated and untreated, control and transgenic, plants were used to evaluate cell viability using a 2,3,5-triphenyltetrazolium chloride (TTC) assay (Hema et al., 2014). Leaf fragments were carefully rinsed in sterile water and kept in TTC solution in the dark for 6 h at room temperature (RT). Leaf samples were then boiled in 5 ml of 2-methoxy ethanol until dry, to release bound formazan. Then, 5 ml of 2-methoxy ethanol was added for the second time, and the change in absorbance was measured at 485 nm.

### Membrane stability index (MSI) and electrolyte leakage (EL)

Healthy fresh leaves were harvested (from treated and untreated; control and *SbSRP* transgenic plants) and MSI was determined by the process explained by Sairam (1994). Leaf segments were kept in sterile water, and 1st set (L1) was stored at 40°C for 30 min where 2nd set (L2) was stored at 100°C for 10 min. Electrical conductivity (EC) of samples from both sets was measured, and MSI was calculated as *MSI* = 100 × 1−*L*1/*L*2.

For analyzing the electrolyte leakage, leaf samples were thoroughly rinsed with sterile water to remove surface bound electrolytes. Fresh leaf segments were stored in the sterile water in sealed tubes and incubated, overnight, on a rotating shaker. The next day, EC was determined (Lt) by a conductivity meter (SevenEasy, Mettler Toledo AG 8603, Switzerland). Then, leaf samples from the each treatment were autoclaved at 120°C for 15 min, subsequently, they were chilled to RT and EC (L0) was determined again (Lutts et al., 1996). The percentage EL was determined as *EL*(%) = 100 × *Lt*/*L*0.

### Quantification of proline, H_2_O_2_, and lipid peroxidation

The free proline and H_2_O_2_ content in the treated and untreated, control, and transgenic plants were calculated by the methods described by Bates et al. (1973) and He et al. (2005), respectively. A standard curve was generated with the known concentrations of proline and H_2_O_2_, an increase in absorbance was recorded at 520 and 560 nm, respectively. Lipid peroxidation was calculated by quantifying the level of MDA (malondialdehyde) produced by TBA (thiobarbituric acid) reaction (Draper and Hadley, 1990).

### Estimation of total soluble sugar, reducing sugar, oligo sugar, free amino acid, and polyphenol content

The total quantities of oligo and reducing sugars were determined by reference to a standard curve that was generated using glucose (Sigma-Aldrich, USA). The treated and untreated, control and transgenic leaf segments were crushed in 85% ethanol, following incubation with an anthrone reagent. All samples were boiled then cooled to room temperature, and absorbance was recorded at 620 and 540 nm. The level of reducing sugar was computed using a colorimetric test and the DNS method (Yemm and Willis, 1954). Total amino acids and polyphenol content were calculated by reference to a standard curve, which was generated by glycine and catechol solution, respectively (Pandey et al., 2015; Patel et al., 2016b). Fresh leaf samples were crushed in 85% ethanol, evaporated to dryness following re-dissolution in ninhydrin or the Folin-Ciocalteu reagent. Variation in color intensity was then analyzed at 570 or 650 nm.

### *In vivo* localization of H_2_O_2_, superoxide-radicals, and starch

The hydrogen peroxide, superoxide-radicals, and starch under salt (200 mM NaCl) or osmotic (10% PEG) stress were determined *in vivo* using a histochemical stain of 3,3-diaminobenzidine (DAB), nitro-blue tetrazolium (NBT), or potassium iodide (KI), respectively (Shi et al., 2010). DAB and NBT were prepared as 1 mg ml^−1^ solutions in 10 mM phosphate buffer (pH 7.8), whereas KI (1 mg ml^−1^) solution made in distilled water. Fresh leaf segments (of treated and untreated, control, and transgenic plants) were dipped in the freshly prepared stain solutions, kept in the dark for 2 h followed by exposure to white light (40 μmol m^−2^ s^−1^ spectral flux photon) for DAB (8 h) and NBT (1 h). For *in vivo* localization of starch, leaf segments were immersed in KI solution for 1 min. The blue or brown spots that appeared on the leaves indicated *in vivo* localization.

### Analyses of ion content

The plant samples (treated and untreated, control, and transgenic plants) were dried at 65°C for 72 h and their dry weights were determined. Plant samples were acid digested overnight in perchloric acid-nitric acid solution (3:1), heated to dryness following re-dissolution in sterile water. Ion content was determined using an inductively coupled plasma optical emission spectrometer (Optima2000DV, PerkinElmer, Germany).

### Quantitative real-time PCR (qRT-PCR) analysis

Total RNA was extracted from control and treated plant samples (WT, VC, and transgenic lines) and cDNA was synthesized using a Superscript RT III cDNA synthesis kit (Invitrogen, USA). A real-time qRT-PCR analysis was performed for antioxidant and transcription factor genes of the host plant (*NtSOD, NtAPX, NtCAT, NtDREB*, and *NtAP2*). A melt curve analysis was also implemented to validate the specificity of the qRT-PCR reaction (Table S1). The housekeeping gene β-tubulin was used as a reference and the relative expression change (compared to WT and control condition) was calculated using the CT method (Livak and Schmittgen, 2001; Schmittgen and Livak, 2008).

### Measuring photosynthesis and respiration

Photosynthesis was computed by determining the rate of change of CO_2_ over time in a leaf covered with a moderately big chamber, following the manufacturer's instructions (LI-6400XTv6, LI-COR Biosciences Inc., Nebraska). The treated (NaCl 200 mM and PEG 10%) and untreated plants of control and transgenic lines were kept under controlled conditions for 24 h and then chlorophyll fluorescence was computed. Photosynthesis, photochemical efficiency, and water use efficiency were investigated. All measurements were taken on four fully expanded leaves. The ratio of variable to maximum fluorescence was then determined as *Fv*/*Fm* = (*Fm* − *F*_0_/*Fm*). The computed light was switched on and actinic light was given for 30 s at an intensity of ~1,000 μmol photons m^−2^ s^−1^ PAR (photosynthetically active radiation) and Fm′ was calculated.

### Statistical analysis

Data from 3 replicates, each containing 15 plants, were documented for each set of experiments. Results are expressed as mean ± standard error. Statistical significance was determined by ANOVA (analysis of variance) amongst the means of WT, VC, and transgenic plants. A Tukey HSD test was used for comparisons and the differences were considered to significant when *p* < 0.05 and these differences are designated by different letters.

## Results

### *In-silico* analysis predicts that the *Sb*SRP may be a novel transporter

The full-length *SbSRP* gene (1262 bp; gene accession no. KX242114) contained a 5′ untranslated leader sequence (5′-UTR: 1–294 bases), an open reading frame (ORF: 753 bp; 295–1047), 3′-UTR (215 bp; 1048–1262) and a poly A tail of 19 base pairs (Figure S1). The *SbSRP* ORF (753 bp) encoded a protein of 250 amino acid residues (Figure S1) with an estimated molecular mass of 80–85 KDa and theoretical isoelectric point (pI) of 8.34. The total number of negatively charged residues (aspartate and glutamate) is 28 and there are 30 positively charged residues (arginine and lysine). The N-terminal of *Sb*SRP is M (methionine) and its estimated half-life is 30 h for mammalian reticulocytes (*in vitro*), >20 h for yeast (*in vivo*), and >10 h for *E. coli* (*in vivo*). The instability index of the *Sb*SRP protein was computed to be 34.52; this classifies the protein as stable according to ExPasy's ProtParam Tool (SIB, Swiss Institute of Bioinformatics). The TMpred program predicts membrane-spanning regions and their respective orientation (Figure S2). The prediction was made using an amalgamation of several weight matrices for the scoring of TMbase, a database of naturally occurring transmembrane proteins. The prediction of the topology of the membrane spanning domains revealed the presence of one transmembrane domain score from 25 to 44 at the N-terminal region (Figure S2). The predicted secondary structure of the deduced *Sb*SRP protein contained topological arrangements of elements (coil, α-helices, and β-strand) with classical folding (Figure S2). The *TrSSP* program predicts that *Sb*SRP is a transporter. The conserved domain database (CDD) categorized the deduced protein as part of the rubber elongation factor/stress related protein particle like superfamily (REF/SRPP). Phylogenetic analyses revealed that *Sb*SRP showed proximity with the *Beta vulgaris* and *Vitis vinifera* hypothetical or predicted REF/SRPP like particle. The *SbSRP* gene sequence showed 78% identity with *Beta vulgaris* subsp. *vulgaris* predicted REF/SRPP like protein *At*3g05500 mRNA, 71% with *Populus trichocarpa* REF family protein, 71% with *Populus trichocarpa* unknown clone mRNA, 71% with *Populus euphratica* REF/SRPP like proteins *At*3g05500 mRNA, 70% with *Ricinus communis* REF like protein, 69% with *Citrus sinensis* predicted REF/SRPP like protein *At*3g05500 mRNA, 69% with *Citrus clementina* hypothetical protein mRNA, and 69% with *Vitis vinifera* predicted SRP like mRNA hence was considered uncharacterized.

### The *Sb*SRP is a plasma membrane localized protein

**Figure 1:**
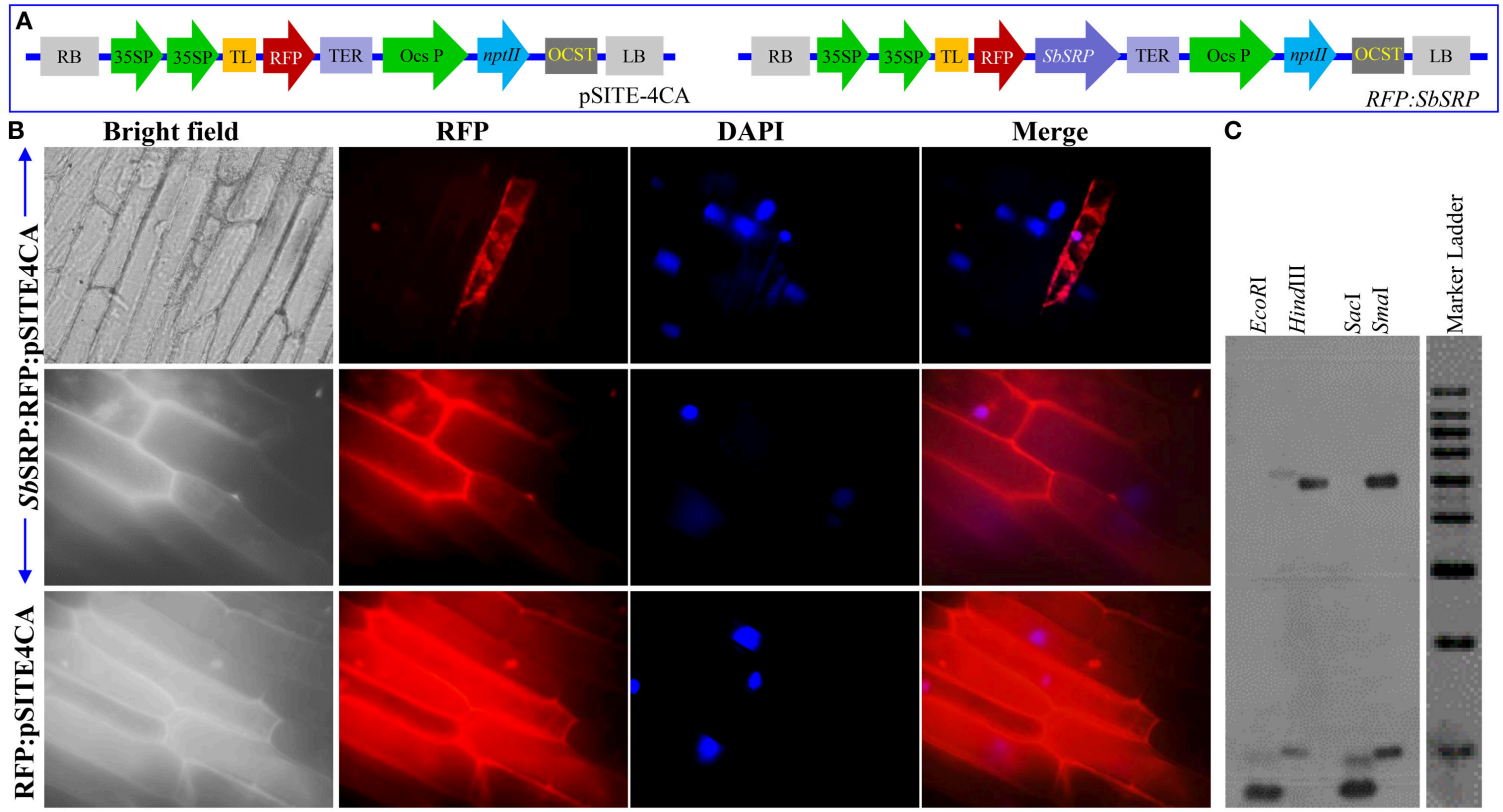
**Sub-cellular localization of *Sb*SRP protein and gene copy number. (A)** Vector *pSITE-4CA*(RFP) and *SbSRP-1:RFP* gene construct, **(B)** Transient expression of RFP alone and *Sb*SRP:RFP translational fusion protein on transformed onion epidermal cells and **(C)** DNA hybridization of *Salicornia brachiata* genome to determine the copy number of *SbSRP* gene.

The transient expression of RFP only and *SbSRP*:RFP (Figure 1A) in onion epidermal cells revealed that *SbSRP* is expressed in the plasma membrane whereas RFP is expressed uniformly throughout the cells (Figure 1B). The sequence analysis of the cDNA clone compared to the genomic clone revealed a single exon structure of *SbSRP* gene, confirming the intronless genomic organization of the gene. In the DNA gel blot analysis, *EcoR*I, *Hind*III, *Sac*I, and *Sma*I-digested genomic DNA produced two intense bands; hence, the existence of at least two homologs of the *SbSRP* gene was depicted (Figure 1C).

### Transcript profiling confirms the differential expression of the *SbSRP* gene

The transcript profile of *SbSRP* was studied in *Salicornia* under salt, desiccation, heat, and cold stress using qRT-PCR (Figure S3). A 3.7-fold exponential increase was observed in the *SbSRP* transcript under 250 mM salt stress; when the salt concentrations was increased further to 500 and 1,000 mM, there was a reduction in the transcript level, and about 1.7 and 1.5-fold expression were detected respectively. Therefore, 250 mM NaCl was found to be the optimum concontration for *SbSRP* transcript expression. Consequently, plants were subjected to 250 mM salt stress for a range of durations from 2 to 24 h, and an exponential increase in the mRNA level of *SbSRP* from 2.5 to 6.5-fold was observed up to 12 h, thereafter expression decreaded and reached to 3.5-fold at 24 h compared to control condition. Under cold and desiccation stresses, relative expression of *SbSRP* transcript was maximum 8.6 and 3.8-fold, respectively at 12 h. Under heat, the *SbSRP* transcript reached a maximum of 25.63-fold at 24 h (Figure S3).

### Analysis confirms overexpression of the *SbSRP* gene in transgenic tobacco lines

Putative transgenic lines (T1) were screened by germination on hygromycin (20 mg L^−1^) and the presence of transgenes was confirmed by PCR amplification of *uid*A and *hptII* genes (Figures S4A,B). Acclimatized putative *SbSRP* over-expressing transgenic lines were confirmed for the stable integration of the transgene into the tobacco genome by DNA hybridization (Figure S4C). Amplification of the *uid*A and *hptII* genes was observed in all the transgenic lines (L1–L17) and the positive control, whereas no amplification was observed in WT (wild type, non-transgenic) plants. DNA gel blot analysis revealed that transgenic lines L3, L6, and L12 had two copies of the transgene, whereas L2, L7, L10, L16, and L17 clearly showed a single transgenic event, validating the single-copy integration of T-DNA into the tobacco genome. All other lines showed faint or unclear transgenic events while WT and VC plants did not illustrate any detectable hybridization signal. Therefore, the comparative ectopic expression analysis of lines L2, L7, L10, L16, and L17 was carried out by semi-quantitative RT-PCR and real-time (qRT) PCR (Figures S5A,B). Transgenic lines L2, L7, and L16 showed 4.6, 5.9, and 5.6-fold increases whereas L10, L12, and L17 showed about 3–4-fold increases under salinity stress. The *SbSRP* over-expressing transgenic lines L2, L7, and L16 exhibited higher expression than the others lines, and no amplification was observed from the cDNA of WT and VC plants. The stringency of the PCR was checked by amplifying the β-tubulin as the internal reference gene using the same cDNA and PCR conditions. An amplicon of ~300 bp was obtained in all transgenic lines and the WT and VC plants. Semi-quantitative RT-PCR and qRT-PCR additionally confirmed the stable integration and expression of the transgene in putative transgenic lines. Transgenic lines (L2, L7, and L16) and control plants (WT and VC) were selected for further analysis. The activity of the reporter gene, β-glucuronidase, in the leaf segment of transgenic, VC and WT plants was visualized, and efficient GUS activity was observed (Figure S5C).

### Overexpression of the *SbSRP* gene enhances plant growth

**Figure 2:**
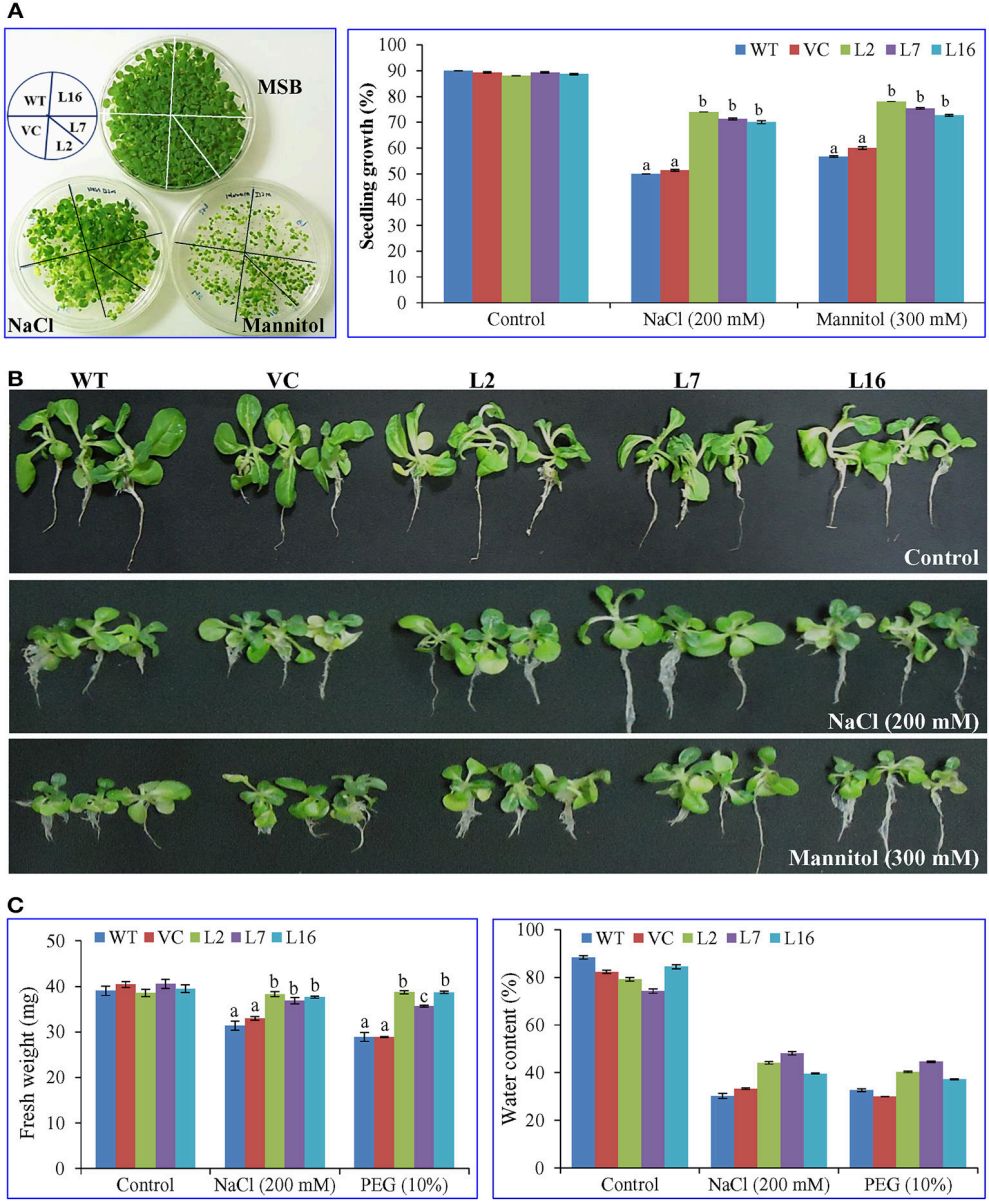
**Plant growth analyses of transgenic tobacco plants under abiotic stress. (A)** Calculation of seedling growth percentage after 21 days. Comparative study of **(B)** morphology and **(C)** fresh weight and water content of transgenic lines (L2, L7, and L16) and control plants (WT and VC), grown for 21 days under salt and osmotic stress. Bars represent means ± *SE* and values with different letters are significant at *P* < 0.05.

The seeds from WT, VC, and transgenic lines (L2, L7, and L16) were harvested from the greenhouse, surface-sterilized, and germinated under different conditions. The *SbSRP* over-expressing transgenic lines L2, L7, and L16 showed ~75% seedling growth under NaCl and osmotic stress conditions (Figure 2). This seedling growth percent was significantly different (*p* < 0.05) to that observed in WT and VC, which exhibited ~54% seedling growth. After 3 weeks of salinity (NaCl 200 mM) and osmotic stress (mannitol 300 mM) treatment, transgenic lines illustrated phenotypically better tolerance than WT and VC plants. Likewise, growth parameters were significantly (*p* < 0.05) enhanced in *SbSRP* over-expressing transgenic lines compared to WT and VC plants under stress conditions (Figure 2).

**Figure 3:**
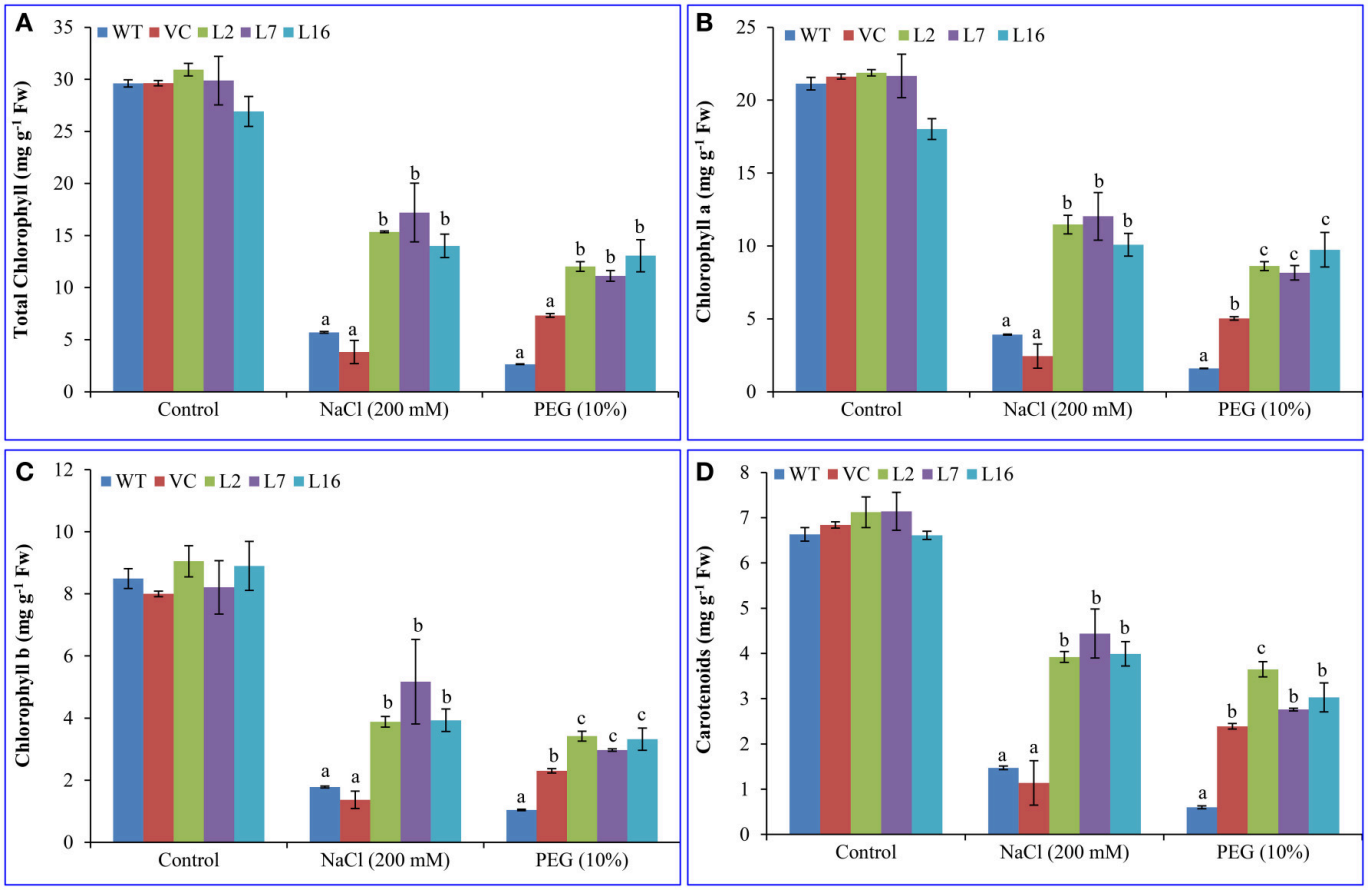
**Estimation of chlorophyll content and carotenoids. (A)** Total chlorophyll, **(B)** chlorophyll a, **(C)** chlorophyll b, and **(D)** carotenoids contents of transgenic (L2, L7, and L16) and control plants (WT and VC) under salt and osmotic stress condition. Bars represent means ± *SE* and values with different letters are significant at *P* < 0.05.

After 7 days of incubation in control conditions, NaCl (100, 150, 200 mM) or PEG (10 and 20%), leaf discs of *SbSRP* over-expressing transgenic lines showed lower degradation of chloroplasts and stayed greener than WT and VC leaf discs (Figure S6A). The total chlorophyll (Chl; about 29.5 mg g^−1^ Fw) and carotenoid (about 6.8 mg g^−1^ Fw) content were almost comparable between control (WT and VC) and transgenic plants under normal (unstressed) conditions (Figure 3). After NaCl and osmotic stress, the total Chl content was reduced ~80%. About 5.7 and 2.6 mg g^−1^ Fw Chl contents were observed in WT under NaCl and osmotic stress, respectively. Similarly, about 3.8 and 7.3 mg g^−1^ Fw Chl were detected in VC plant under NaCl and osmotic stress, respectively. The degree of reduction was less in transgenic lines and about 13.8 and 3.6 mg g^−1^ Fw total Chl and carotenoid contents were quantified, respectively. Transgenic lines showed better protection of Chl from salt and osmotic stress conditions than WT and VC plants. Individually, transgenic lines, L2, L7, and L16, showed about 50% (30.94–15.35 = 15.59 mg g^−1^ Fw), 42% (29.88–17.21 = 12.67 mg g^−1^ Fw) and 48% (26.92–14.01 = 12.91 mg g^−1^ Fw) reduction under salinity (NaCl) stress. Similarly, a 61% (30.94–12.03 = 18.91 mg g^−1^ Fw), 63% (29.88–11.12 = 18.76 mg g^−1^ Fw) and 52% (26.92–13.06 = 13.86 mg g^−1^ Fw) reduction was found in transgenic lines (L2, L7, and L16) under osmotic stress, respectively. Total carotenoid content also exhibited a similar pattern as chlorophyll content (Figure 3). About an average of 47% (6.8–3.6 = 3.2 mg g^−1^ Fw) decrease was observed in transgenic lines compared to 79.5% (6.8–1.4 = 5.4 mg g^−1^ Fw) reduction in control plants under stress conditions. Furthermore, transgenic lines showed significantly (*p* < 0.05) improved cell viability in contrast to WT and VC plants under salt and osmotic stress conditions (Figure S6B).

### Overexpression of the *SbSRP* gene improves physiology of the plant

**Figure 4:**
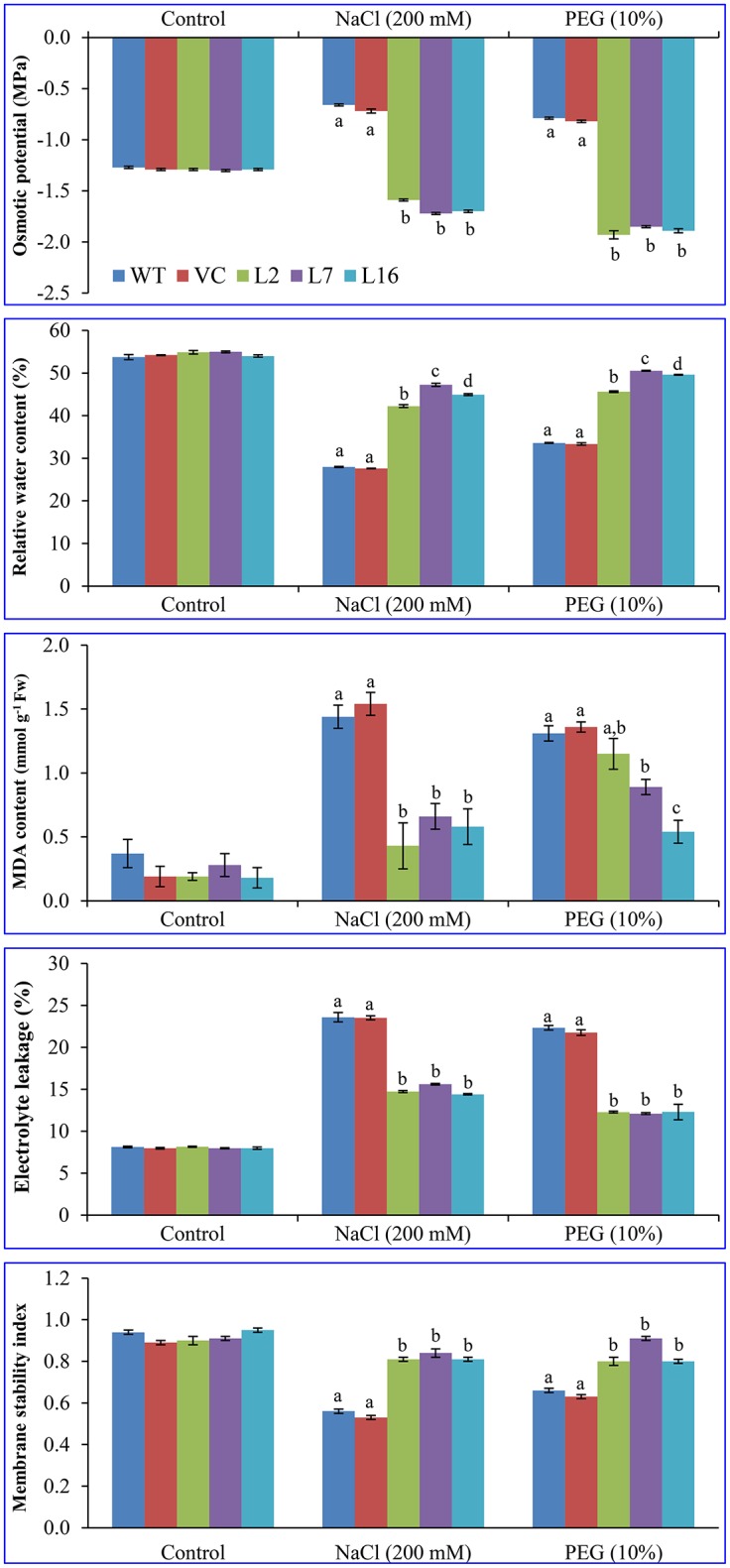
**Physiological analyses of transgenic lines**. Estimation of osmotic potential (OP), relative water content (RWC), lipid peroxidation (MDA content), electrolyte leakage (EL), and membrane stability index (MSI) from leaves of control plants (WT and VC) and transgenic lines (L2, L7, and L16) under control, salinity and osmotic stress conditions. Bars represent means ± *SE* and values with different letters are significant at *P* < 0.05.

Osmotic potential and water retention ability of the WT, VC, and transgenic plant leaves were evaluated under control, salinity, and osmotic stress conditions (Figure 4). All transgenic lines were better able to adjust osmotically (−1.7 to −1.9 MPa) under both salinity and osmotic stress conditions compared to WT and VC plant (−0.6 to −0.8 MPa) leaves. The relative water content (RWC) of all transgenic lines was significantly higher under stress conditions than that of WT and VC plants during salinity and osmotic stress. At control condition, an average of 55% RWC was observed in all plants. About 42–50% RWC was detected in transgenic lines under stress conditions compared to control plants (28–33% RWC in WT and VC). Lipid peroxidation of WT, VC, and transgenic lines was compared in leaf segments by estimating the MDA content (about 0.24 mmol g^−1^ Fw), which is generated after lipid peroxidation and is accumulated in leaves. The MDA content abruptly increased (about 6-fold) in the control (WT and VC) plants under stress, and 1.38 and 1.45 mmol g^−1^ Fw MDA contents were detected in WT and VC, respectively. The MDA content also increased in *SbSRP* over-expressing transgenic lines by 1.8–2.7-fold, and 0.43, 0.66, and 0.58 mmol g^−1^ Fw MDA contents were detected in transgenic lines, L2, L7, and L16, respectively, under salinity stress. Similarly, 2.2–4.7-fold increase was observed under osmotic stress, and 1.15, 0.89, and 0.54 mmol g^−1^ Fw MDA contents were measured. Electrolyte leakage (%) from the leaves of WT, VC, and transgenic lines were not significantly different and was approximately an average of 8% in control conditions. Under stress conditions, the leakage in WT and VC plants increased 2.9-fold, and approximately an average of 23% EL was observed in comparison to that of the control. The electrolyte leakage in *SbSRP* over-expressing transgenic lines (13.5%) increased about 1.7-fold upon stress treatment, but it was significantly lower (23.0–13.5 = 9.5%) than that of WT and VC plants (i.e., 23%). In control conditions, the membrane stability index (MSI) was comparable (about 0.92) in all plants (WT, VC, and transgenic lines). Transgenic lines showed a significantly (*p* < 0.05) higher MSI (about 0.83) under stress conditions compared to control plants (0.59; WT and VC). Results suggest that transgenic plants maintained membrane stability about 1.4-fold better under stress condition than that of WT and VC plants.

### The *SbSRP* gene involves in ion homeostasis

The Na^+^ content under control conditions was almost comparable (0.55 mg g^−1^ Dw) in WT, VC and transgenic lines but found lower under in transgenic lines (compared to WT and VC) under stress conditions. During salinity stress, Na^+^ content in WT and VC plants increased about 1.1-fold; transgenic lines also accumulated Na^+^, but not to the same extent as WT and VC plants (Figure S7). The K^+^ content under control conditions was similar (about 0.4 mg g^−1^ Dw) in all plant types, but WT and VC showed more than a 60% reduction in K^+^ content (0.4–0.15 = 0.25 mg g^−1^ Dw) under salinity stress, in comparison to transgenic lines (0.4–0.26 = 0.14 mg g^−1^ Dw) where the reduction was only 35%. Likewise, the K^+^/Na^+^ ratio was found to be significantly higher in transgenic lines over-expressing the *SbSRP* gene (Figure S7).

### Overexpression of the *SbSRP* enhances biochemical status of the plant

**Figure 5:**
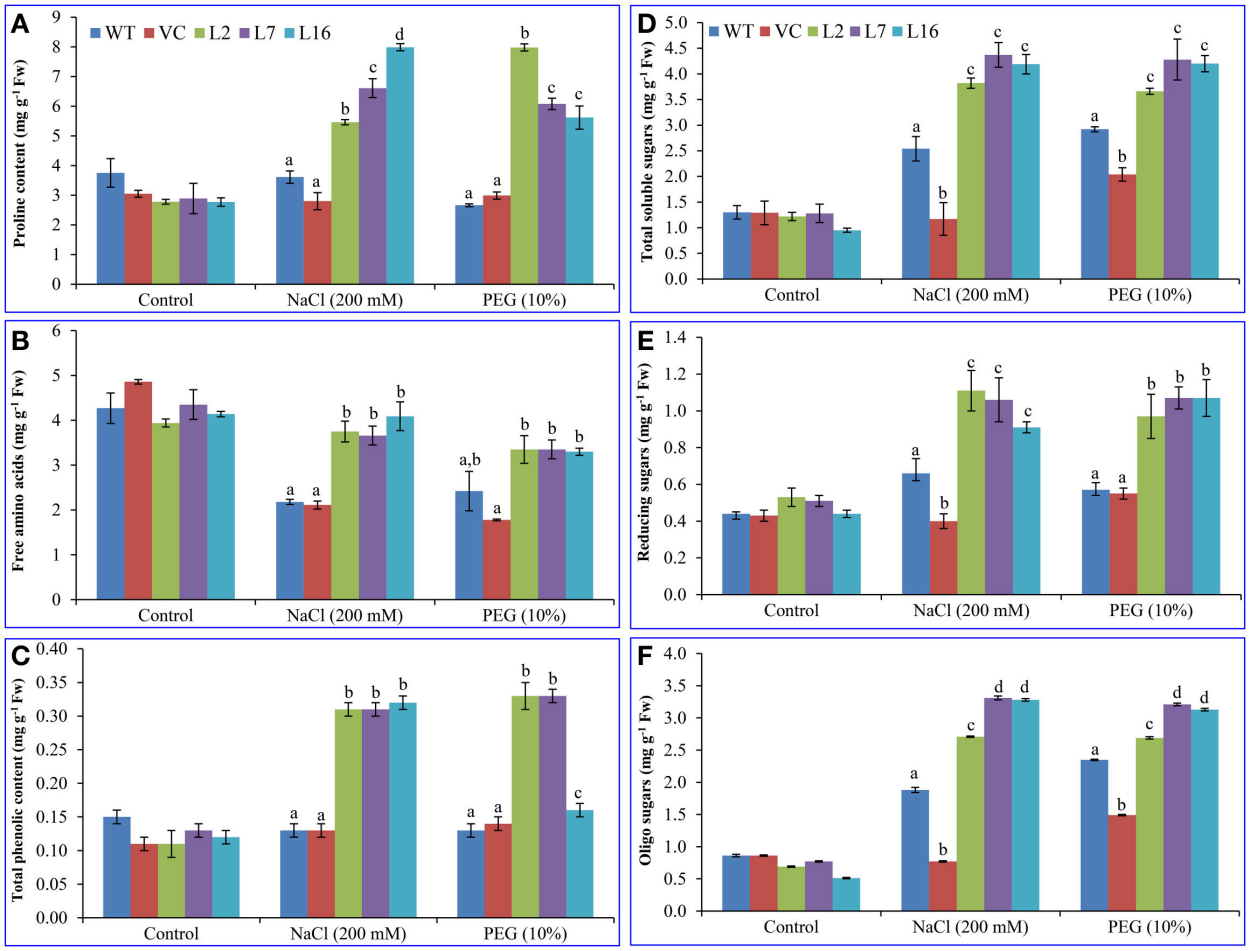
**Biochemical analyses of transgenic tobacco plants under abiotic stress**. Estimation of **(A)** proline, **(B)** free amino acids (FAA), **(C)** total phenolic content (polyphenol; TPC), **(D)** total soluble sugars (TSS), **(E)** reducing sugars (RS), and **(F)** oligo sugars (OS) in control (WT and VC) and transgenic plants (L2, L7, and L16) under salinity and osmotic stress condition. Bars represent means ± *SE* and values with different letters are significant at *P* < 0.05.

No significant difference was observed in the proline (~3.05 mg g^−1^ Fw), free amino acid (~4.32 mg g^−1^ Fw), and polyphenol (0.12 mg g^−1^ Fw) contents between control (WT and VC) and transgenic lines, over-expressing *SbSRP* gene, under control condition (Figure 5). Proline content increased about 1.5–2-fold under salinity stress (5.46, 6.61, and 7.99 mg g^−1^ Fw) and osmotic stress (5.2, 3.19, and 2.85 mg g^−1^ Fw) conditions in transgenic lines L2, L7, and L16, respectively, while WT and VC plants showed a non-significant increase (about 0.03 mg) in proline content (Figure 5A). Transgenic plants maintained the free amino acid content (about an average of 3.6 mg g^−1^ Fw) under stress conditions in contrast to WT and VC (2.1 mg g^−1^ Fw) plants (Figure 5B). The polyphenol content was increased about 2.6-fold (~ 0.32 mg g^−1^ Fw) in all transgenic lines under stresses, except L16, which showed a different result and accumulated only 0.04 mg (about 1.3 times) polyphenol than control plants (WT and VC) under osmotic stress (Figure 5C).

Total soluble sugars (about an average of 1.2 mg g^−1^ Fw) increased about 3.5–4.5-fold under salinity and osmotic stress conditions in transgenic lines (about an average of 4.1 mg g^−1^ Fw) as compared to the control conditions, but only a slight change (1–2-fold) was observed in control (WT and VC; about an average of 2.2 mg g^−1^ Fw) plants (Figure 5D). The reducing sugar level (about an average of 0.47 mg g^−1^ Fw in control condition) in transgenic plants increased 2.2-fold (1.03 mg g^−1^ Fw) under salinity and drought stress conditions whereas a slight increase (about 1.2-fold) was observed in WT and VC (about an average of 0.55 mg g^−1^ Fw) plants (Figure 5E). The oligo-sugar content (about an average of 0.74 mg g^−1^ Fw) was comparable under control conditions, whereas transgenic lines L7 and L16 exhibited about 4.4-times higher oligo-sugar content (about an average of 3.23 mg g^−1^ Fw) under stress conditions (Figure 5F).

### *In-vivo* localization and estimation of ROS

**Figure 6:**
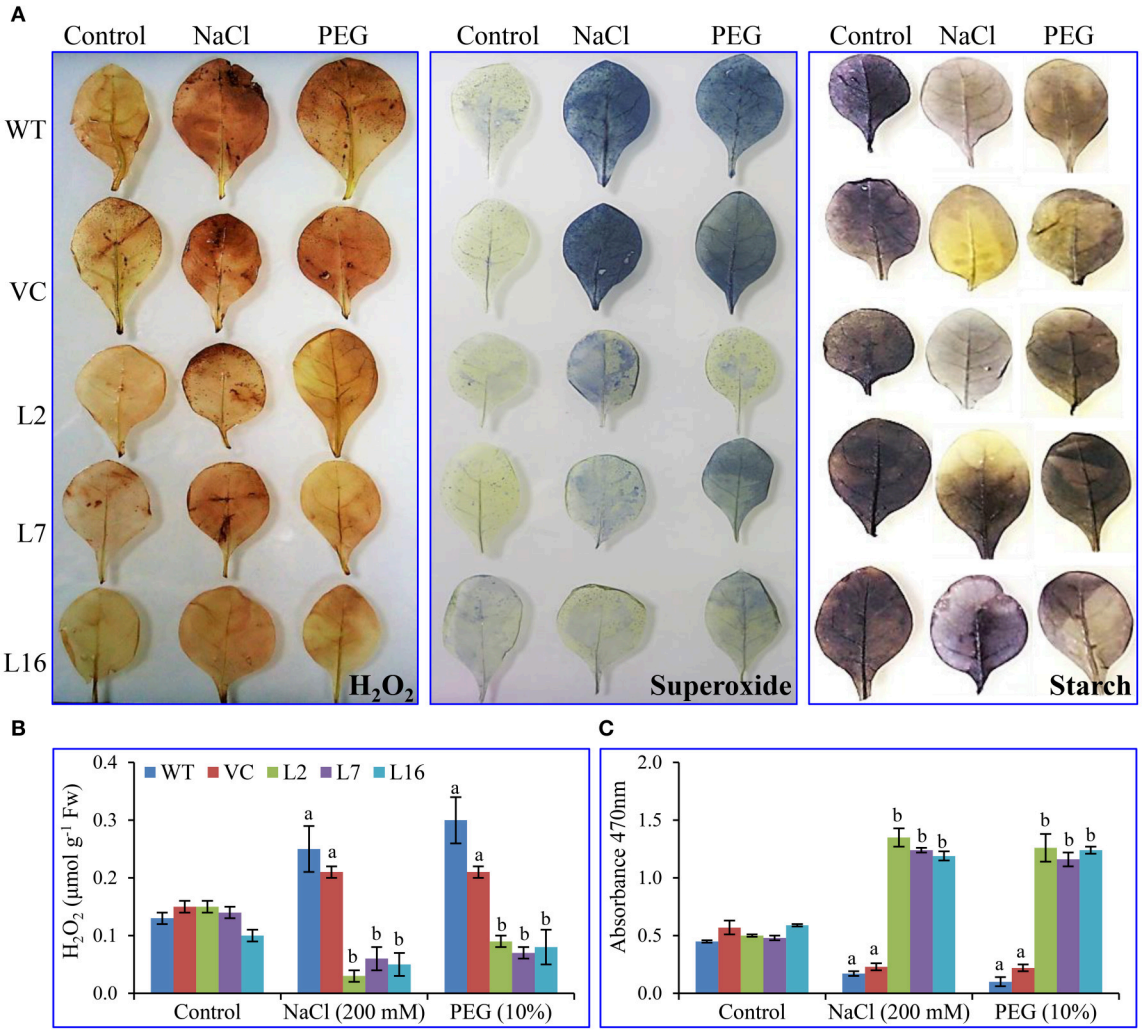
***In vivo* localization and cell viability. (A)** *In vivo* localization of H_2_O_2_, superoxide and starch, estimation of **(B)** H_2_O_2_ content and **(C)** superoxide radicals of transgenic (L2, L7, and L16) and control (WT and VC) leaves. Bars represent means ± *SE* and values with different letters are significant at *P* < 0.05.

Fresh leaf segments (of treated and untreated, control and transgenic plants) were dipped in the freshly prepared DAB and NBT stain solutions, kept in the dark for 2 h followed by exposure to white light for *in-vivo* localization study. There was no variation in peroxide localization or free radicals among leaves of WT, VC, and *SbSRP* over-expressing transgenic lines under control conditions following staining with DAB and NBT (Figure 6A). In contrast, WT and VC plants leaves displayed elevated levels of brown and blue insoluble precipitate formation in comparison to transgenic lines under salt and osmotic stress. These results demonstrate that WT and VC leaves build up more superoxide-radicals and H_2_O_2_ than those of *SbSRP* over-expressing transgenic lines under stress, confirming that *SbSRP* mitigates abiotic oxidative stress *in situ*. Similarly, the cellular starch content was equivalent under control conditions, but transgenic lines showed a deep purple stain under salt and osmotic stress conditions, showing that *SbSRP* over-expressing transgenic lines maintained cellular starch content, even under stress conditions.

Lower accumulation of ROS was observed in transgenic lines (an average of 0.063 μmol g^−1^ Fw), whereas WT and VC plants exhibited the higher accumulation of H_2_O_2_ (an average of 0.25 μmol g^−1^ Fw) under salt and osmotic stress conditions (Figure 6B). The superoxide content was measured by the reduction of XTT tetrazolium salt, where the transgenic lines showed a significant (*p* < 0.05) increase in absorbance (470 nm) in contrast to WT and VC plants under stress conditions (Figure 6C).

### The *SbSRP* gene may regulate the transcript expression of antioxidative enzyme-encoding and transcription factor genes

**Figure 7:**
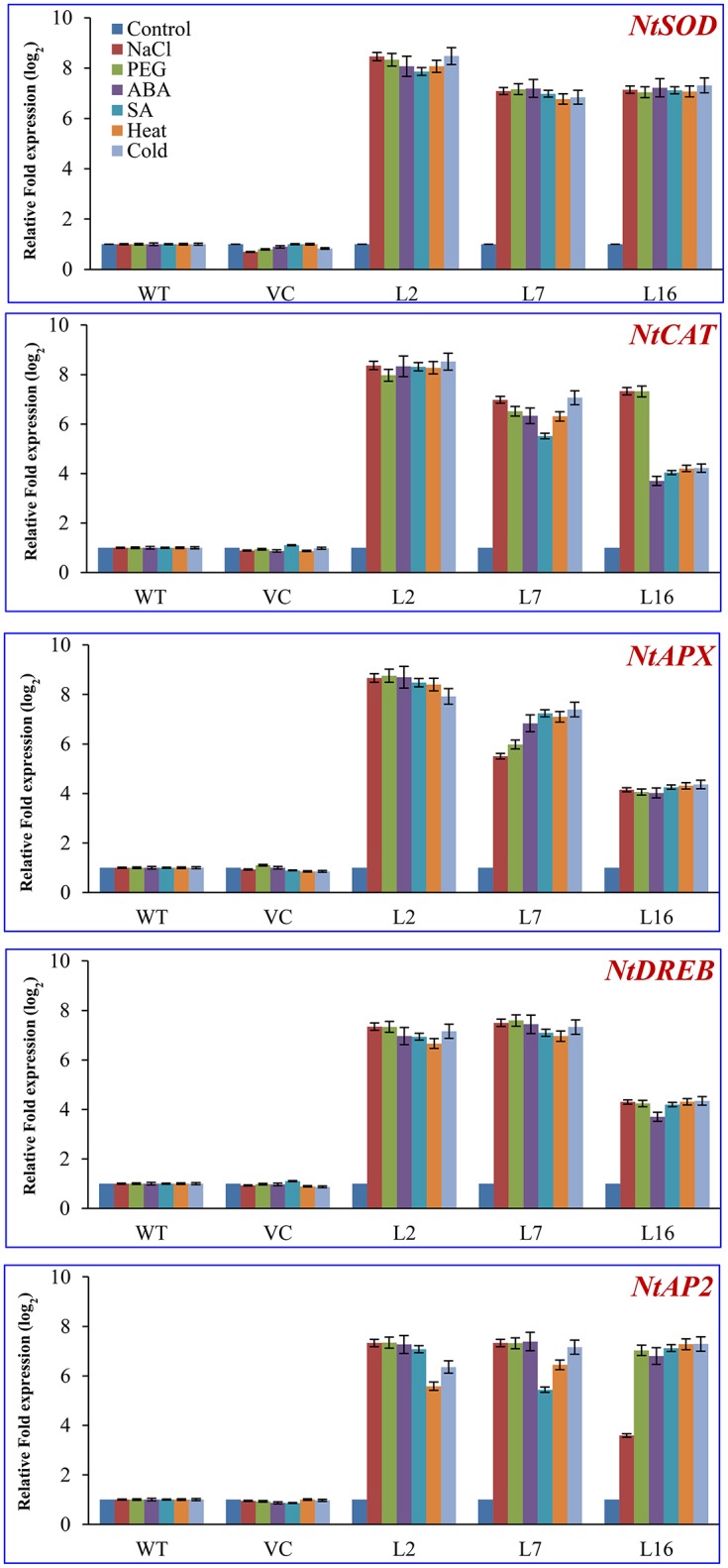
**Transcript expression analysis of host stress responsive genes**. Comparative transcript expression profile of antioxidant and transcription factor genes of the host plant (*NtSOD, NtAPX, NtCAT, NtDREB*, and *NtAP2*) under different stress conditions. The fold expression values obtained in WT (control or stress) were taken as one, relative expression change was calculated for VC and transgenic lines, and presented as relative fold expression (in log_2_). The housekeeping gene β-tubulin was used as a reference.

The mRNA level of genes encoding antioxidative enzymes and transcription factors (ascorbate peroxidase [*Nt-APX*], catalase [*Nt-CAT*], superoxide dismutase [*Nt-SOD*], DREB [*Nt-DREB*], and AP2 [*Nt-AP2*]) were studied in WT, VC, and the transgenic lines (L2, L7, and L16) under control and stress conditions (salinity, osmotic, heat, cold, ABA, and SA; Figure 7). The expression of *Nt-APX* was upregulated by more than 8-fold under salt, drought, ABA, SA, heat, and cold stress in L2, whereas an ~5–7-fold increase was observed in L7 under stress compared to WT, VC plants, and control condition. However, L16 showed lower accumulation of the transcript of *Nt-APX* under stress conditions. The *Nt-CAT* gene transcript also showed a similar pattern, although L16 accumulated about 8 times more transcript under salt and drought stress than WT and VC plants. The transcript of *Nt-SOD* increased by 8-fold in L2, and by 7-fold in L7 and L16 under stress conditions. Likewise, the transcript of *Nt-DREB* and *Nt-AP2* accumulated 4–6 times more in transgenic lines compared to WT and VC plants under stress conditions.

### Overexpression of the *SbSRP* gene enhances photosynthesis of the plant

**Figure 8:**
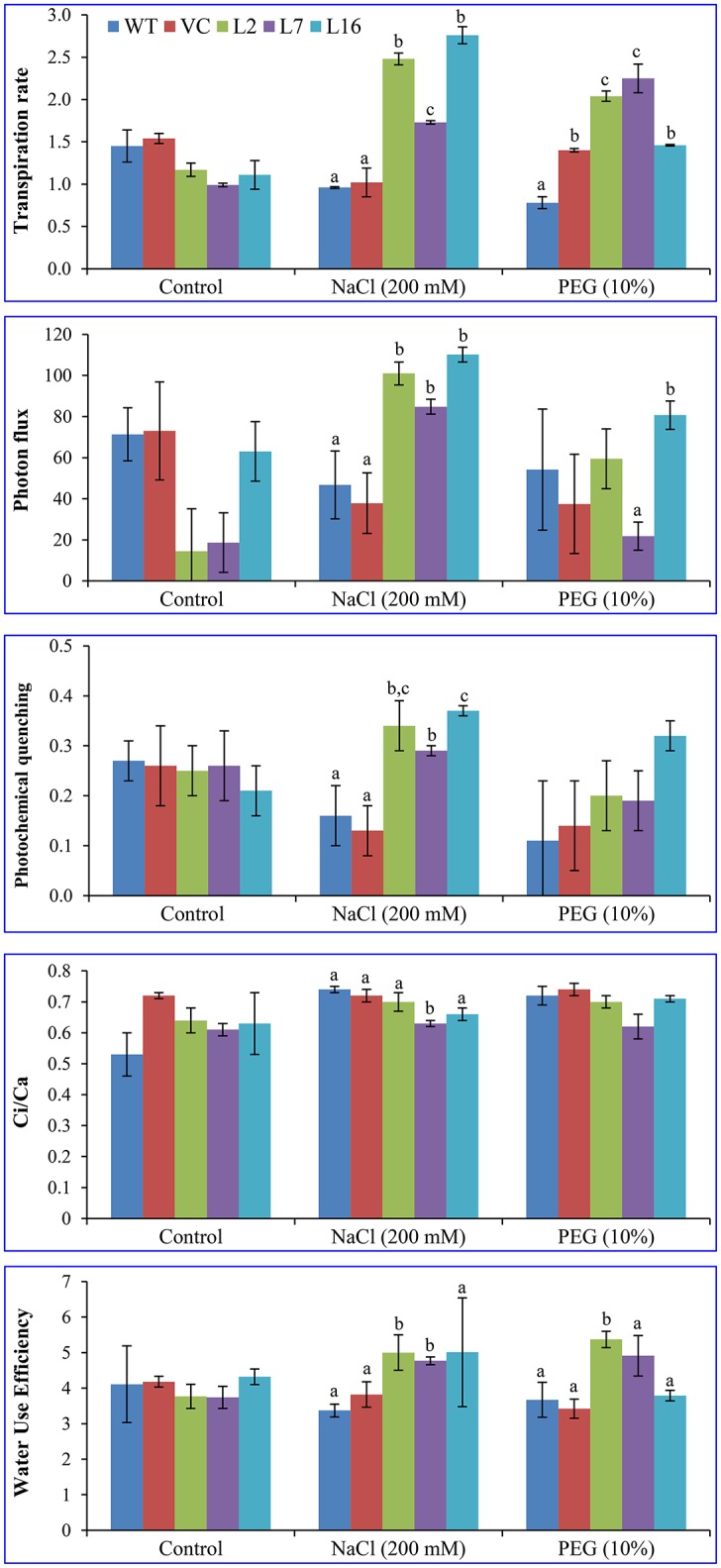
**Quantitative analysis of the physiological status of transgenic lines**. Transpiration rate, photon flux, photochemical quenching, Ci/Ca ratio, and water use efficiency of WT, VC, and transgenic lines (L2, L7, and L16) were measured under salinity and osmotic stress condition. Bars represent means ± *SE* and values with different letters are significant at *P* < 0.05.

Net assimilation rate was ~12 μmol CO_2_ m^−2^ s^−1^ in WT, VC, and transgenic plants under control conditions. Under stress conditions, it reduced to 4 μmol CO_2_ m^−2^ s^−1^ in WT and VC plants, but transgenic plants (L2, L7, and L16) maintained values of 6.5, 8.0, and 5.0 μmol CO_2_ m^−2^ s^−1^, while transpiration rate was 2.0, 1.5, and 2.8 mmol H_2_O m^−2^ s^−1^. These results confirmed the role of *SbSRP* in providing abiotic stress tolerance to the transgenic tobacco plants by maintaining photosynthesis under stress condition (Figure 8).

## Discussion

As plants are sessile in nature, they have to face numerous abiotic stresses such as salt, drought, extreme temperature, variation in light, and UV radiation. Salinity influences almost every aspect of plant physiology, commonly at the entire plant level, through osmotic stress in early stages, and ionic stress at later phases of development (Munns and Tester, 2008). Abiotic stresses, along with population increases and lack of food, create trouble for people globally, and cause losses worth hundreds of million dollars every year. Salinity is developing as a noteworthy issue, and halophytes give a remarkable opportunity to adjust crops to different saline biological niche (Mishra et al., 2015). Halophytes are well-adapted to high salinity due to their modified molecular, biochemical, morphological, anatomical, and physiological characteristics. Halophytes have therefore developed a cascade of genes in order to achieve tolerance, including ion compartmentalization, osmotic adjustment (osmolytes), succulence (ion sequestration in vacuoles), selective uptake and ion transport (*SOS1, NHX1, HKT2, CLC*), enzymatic (SOD, APX, GST, GR) and non-enzymatic (proline, glycine betaine, ascorbate, tocopherol, polyols, polyamines) antioxidant response, maintenance of redox/energetic status, salt inclusion/excretion, and genetic control (DREB, NAC, MYB). The present study reported the isolation, cloning and functional validation of a novel salt-responsive gene, *SbSRP*, from an extreme halophyte *S. brachiata*. The *SbSRP* gene was characterized at the genomic and transcriptomic levels and was genetically transformed into *Nicotiana tabacum* cv. Petit Havana. The role of the candidate *SbSRP* gene under salinity and osmotic stress tolerance was studied in the putative T1 generation of transgenic tobacco.

The full length *SbSRP* sequence was 1,262 bp and included a 5′ untranslated leader sequence (5′-UTR: 1–294 bp), an open reading frame (753 bp ORF that encoded a protein of 250 aa with an estimated molecular mass of 80–85 kDa), a 3′-UTR (215 bp; 1,047–1,262) and a poly A tail of 19 base pairs (Figure S1). The genomic organization of the *SbSRP* gene revealed that it is an intronless gene, consisting of a single exon. Jain et al. (2008) reported that the rice and *Arabidopsis* contain 19.9 and 21.7% intronless genes, respectively. These results may support future work on the role of intronless genes in plants and other higher organisms by comparative, evolutionary, and functional analysis. Yan et al. (2014) demonstrated that maize intronless genes (36.87%) play a crucial role in energy metabolism and translation. Intronless genes are widespread in prokaryotes and are also additionally seen as rapidly inducible eukaryotic genes (Giri et al., 2013). At least two homologs of the *SbSRP* gene are present in the *S. brachiata* genome, as shown by DNA gel blot analysis (Figure 1C). The DNA sample digested with *Hind*III, *EcoR*I, *Sma*I, and *Sac*I showed only two bands, signifying the existence of two copies of the gene.

The *SbSRP* transcript showed higher expression under salt, desiccation, heat, and cold stress in *S. brachiata* (Figure S3). Zhang et al. (2014) stated that transcriptome data could provide insight into the molecular mechanisms behind the response to salt stress in the physic nut and might provide an asset for the genetic engineering of salt tolerance in crops. These results support the notion that *Salicornia* shows enhanced abiotic stress tolerance due to the accumulation of transcripts of several novel salt responsive genes, of which *SbSRP* is one (Jha et al., 2009).

The *SbSRP* gene sequence showed 80% identity with *Beta vulgaris*, 71% with *Populus trichocarpa*, 71% with *Populus euphratica*, 70% with *Ricinus communis*, and 69% with *Citrus sinensis* predicted REF/SRPP-like protein. Transient RFP: *Sb*SRP expression analysis clearly indicated that SRP was preferentially compartmentalized in the plasma membrane (Figure 1). This was further confirmed by *in silico* TMpred analysis, which showed the presence of one transmembrane domain (Figure S2). To reveal the capacity of an unknown gene, subcellular localization studies are another way to identify the function of the relating protein. The area of the reporter gene in a subcellular compartment, as coordinated by the unknown intertwined protein, frequently provides support for identifying the function and confinement of the gene (Curtis and Grossniklaus, 2003). Crosstalk at the plasma membrane interface is imperative for sessile living beings, such as plants, which are required to keep up a various range of plasma membrane receptor and transport proteins so as to have the capacity to sense and react to changes in their surroundings (Luschnig and Vert, 2014). The present study confirms that *Sb*SRP is a plasma membrane protein, which is in line with *in silico* studies (Figure 1 and Figure S2). Based on conserved motifs proposed for a special REF/SRPP-like family of uncharacterized proteins in plants, *Sb*SRP is categorized as an SRP-like protein, which is a stress-related protein that may be released into the cytosol during osmotic lysis. The *SbSRP* gene was transformed and transgenic tobacco plants were developed. Successful integration of the transgene was validated by PCR and DNA gel blot analysis (Figure S4). The semi-quantitative RT-PCR and qRT-PCR showed higher expression of the transgene (Figure S5). Three transgenic lines (L2, L7, and L16) along with control plants (WT and VC) were selected for physio-morpho-biochemical and molecular analysis based on higher gene expression, histochemical GUS expression, and percent seedling growth. Yue et al. (2012) verified that transgenic tobacco plants illustrated higher rates of seed germination and chlorophyll content relative to WT in response to salt stress.

The WT and VC plants did not show any expression of the transgene while the transgenic lines L2, L7, and L16 showed a higher level of *SbSRP* transcript expression than that of other lines although a comparable amount of cDNA was used (Figure S5). Chlorophyll content was considered as one of the indicators of cellular stress, and it reduces in plants during abiotic stress. In the present study, Chl a, Chl b, total chlorophyll, and carotenoid content were considerably decreased in WT and VC plants under salinity or osmotic stress compared to transgenic lines (Figure 3). The differences between chlorophyll contents may vary while considering relative water contents. Similarly, transgenic plants (tobacco and groundnut) over-expressing *SbpAPX* and *SbASR-1* showed elevated chlorophyll content under oxidative stress provoked by osmotic and salinity stress (Singh et al., 2014a,b; Tiwari et al., 2015b). Plant senescence is characterized by the degradation of cellular components, which leads to the loss of compartmentalization and tissue structure, resulting in plant death (Fan et al., 1997). Transgenic plants could withstand higher salt stress than the wild type in leaf disc assays and pots (Joshi et al., 2013; Singh et al., 2016). Based on this evidence, it is presumed that over-expressed *Sb*SRP may have sheltered the chlorophylls from oxidative damage, and consequently, transgenic lines retain elevated chlorophyll, and carotenoid content. Ramel et al. (2012) reported that carotenoids play a key role in the protection of the photosynthetic machinery by stabilizing thylakoid phospholipids, hence reducing the excited triplet state of chlorophyll and singlet oxygen in salinity and osmotic stress conditions.

The RWC analysis of WT and VC plants and transgenic tobacco lines demonstrated higher relative water content in the transgenic lines than in WT and VC plants under salinity and osmotic stress conditions (Figure 4). Similar results were reported by Jha et al. (2011), Chaturvedi et al. (2014), Patel et al. (2015), Tiwari et al. (2015b), and Udawat et al. (2016), where *SbASR-1, SbNHX-1, SbMT-2*, and *SbUSP* over-expressing transgenic tobacco, castor, and groundnut lines showed a lower rate of water loss in salt and drought stress. MDA formation and electrolyte leakage are universal stress indicators that determine the degree of damage caused by stress in plants; stress-induced ROS generation is accountable for these leakages and the accumulation of MDA. MDA is the byproduct of lipid peroxidation due to the production of free radicals during stress, while electrolyte leakage is the release of K^+^ from ROS-activated cation channels against its counter ions Cl^−^, ${\text{HPO}}_{4}^{2-}$, ${\text{NO}}_{3}^{-}$, citrate and malate (Demidchik et al., 2014). The present study reported elevated MDA content and electrolyte leakage in WT and VC plants in comparison to *SbSRP* over-expressing transgenic lines under salinity and osmotic stress conditions (Figure 4). Conversely, under control conditions, all plant types showed a comparable level of MDA and electrolyte leakage.

The cellular stress marker proline was quantified, which accumulates during osmotic stress to balance the osmotic homeostasis across the membrane. In the present study, the *SbSRP* over-expressing putative transgenic lines exhibited higher accumulation of free proline than WT and VC plants under salinity and osmotic stress conditions (Figure 5). Analogous results were observed in transgenic tobacco lines over-expressing *SbUSP* grown under salinity and osmotic stress conditions (Udawat et al., 2016). The accumulation of this amino acid, primarily during salinity and drought stress, is considered to play an important role as an osmoregulatory osmolyte (Delauney and Verma, 1993) and also acts as a signaling molecule (Szabados and Savoure, 2010). Total soluble sugar, reducing sugars, oligo-sugars, and polyphenol content were found to be significantly higher in the transgenic tobacco lines over-expressing the *SbSRP* gene compared to that of WT and VC plants under salinity and osmotic stress conditions (Figure 5). Tobacco *ASR*-1 improved the transcript accumulation of sugar transporters, viz hexose transporter, sucrose transporter and vacuolar glucose transporter proteins (Dominguez et al., 2013). Glucose and sucrose are transported from the leaves to the phloem with the help of these transporters, in order to then mobilize the sugars to other plant organs. Based on our experimental results, we hypothesize that *Sb*SRP may regulate several sugar transporter proteins and improve the abiotic stress tolerance in transgenic plants by the mobilization of plant photosynthate to the root or other non-photosynthetic organs.

Fresh leaf segments (of treated and untreated, control, and transgenic plants) were dipped in the freshly prepared DAB and NBT stain solutions, kept in the dark for 2 h followed by exposure to white light for *in-vivo* localization study. Peroxide and superoxide free radicals were *in vivo* localized in leaves of WT, VC, and transgenic plants (Figure 6). An almost negligible amount of free radicals (insoluble brown and blue-colored precipitate) was observed under control conditions, whereas under salt and osmotic stress, the leaves from the WT and VC plants exhibited higher accumulation of brown and blue-colored precipitate following DAB and NBT histochemical staining. This observation suggests that there is a greater accumulation of peroxide and superoxide free radicals in WT and VC plants under stress conditions. In agreement with our findings, transgenic tobacco and groundnut lines over-expressing *SbpAPX* and *SbASR-1* also exhibited reduced accumulation of H_2_O_2_ content and superoxide-radicals free radicals in leaves grown under salt and drought stress (Singh et al., 2014a,b; Tiwari et al., 2015b). Likewise, leaves of the putative transgenic lines over-expressing *SbSRP* retained a higher amount of starch (observed as a purple precipitate), even under salinity and osmotic stress conditions, in contrast to WT and VC plant leaves. Parvaiz and Satyawati (2008) reviewed carbohydrates such as sugars (glucose, fructose, sucrose, fructans) and found that starch accumulation under salt stress may play a key role in osmoprotection, osmotic adjustment, carbon storage, and radical scavenging. Baena-Gonzalez et al. (2007) described the role of most the important *KIN10* activated genes and their control over the majority of catabolic pathways, including cell wall, starch, sucrose, amino acid, lipid and protein degradation, that supply other sources of energy and metabolites during stress conditions.

To further validate the probable regulatory role of the *Sb*SRP protein, transcript expression of antioxidant-encoding genes *Nt-APX* (cytosolic ascorbate peroxidase), *Nt-CAT* (catalase), *Nt-SOD* (mitochondrial superoxide dismutase), and transcription factor-encoding genes, *Nt-DREB* and *Nt-AP2*, were analyzed in WT, VC, and transgenic plants subjected to control conditions, salinity, drought, ABA, SA, heat, and cold stress (Figure 7). The relative increase in *Nt-APX, Nt-CAT, Nt-SOD, Nt-DREB*, and *Nt-AP2* transcript expression was higher in transgenic lines under stress conditions. Liu et al. (2013) reported that *Arabidopsis* transgenic lines over-expressing *LcASR-1* showed elevated transcript expression of *CAT, SOD, APX*, and glutathione reductase enzyme genes, thus contributing to improve ROS scavenging in transgenic plants.

**Figure 9:**
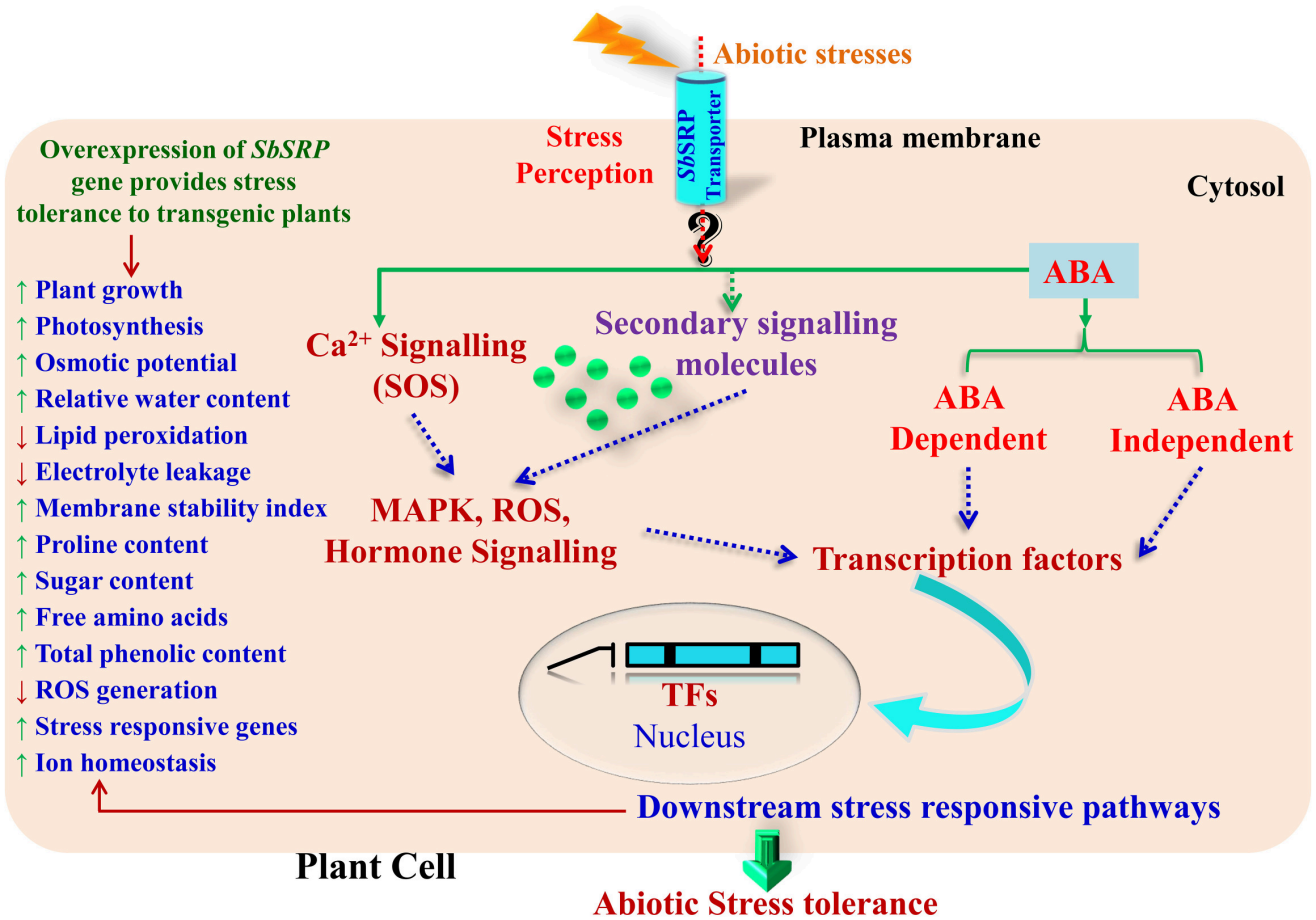
**A hypothetical model to infer the putative role of membrane bound *Sb*SRP-like protein in abiotic stress tolerance mechanism**. The salt tolerance mechanism shown in this model is a schematic representation of a general salt tolerance mechanism (irrespective of organelle-specific) using previous studies.

Based on transcript expression analysis, it was concluded that *SbSDR-1* and *SbUSP* genes (cloned from *S. brachiata*) involve in the transcriptional regulation of antioxidant enzyme-encoding genes (Singh et al., 2016; Udawat et al., 2016). The present study is in agreement with these reports and suggests that *Sb*SRP may be further implicated in ROS scavenging activity directly as a non-enzymatic antioxidant or indirectly by inducing the expression of several genes encoding antioxidative enzymes (Figure 7). Wang et al. (2011) demonstrated that the activity of ascorbate peroxidase (APX) and catalase (CAT), which are two key reactive oxygen species (ROS) detoxifying enzymes, were elevated in transgenic plants exposed to salt stress in comparison to WT plants. The primary job accomplished by APX and CAT is to detoxify the H_2_O_2_ into water molecules, which under normal conditions is kept under control. Similar results were found in transgenic tobacco and groundnut lines over-expressing *SbASR-1* (Chaturvedi et al., 2014; Tiwari et al., 2015b). The main effect of abiotic stresses on plant growth/productivity is correlated with the communication occurs between organs and each cell types have less or more specific response to the stress. Based on results, it is speculated that membrane-localized *Sb*SRP-like protein may regulate the expression of the antioxidative and transcription factor genes; it may sense environmental stimuli and transmits downward through the plasma membrane (Figure 9). The salt tolerance mechanism shown in this model is a schematic representation of a general salt tolerance mechanism (irrespective of organelle-specific) using previous studies.

## Conclusion

In this study, the *SbSRP* gene was cloned from an extreme halophyte *S. brachiata* and functionally characterized using transgenic approach. The genome organization study revealed the two homologs of an intronless *SbSRP* gene. The *in silico* and *in vivo* localization study suggest that the *SbSRP* protein is confined to the plasma membrane. Morphological, biochemical, and physiological analyses of T1 transgenic tobacco lines showed that transgenic plants are more tolerant to salinity and osmotic stress than wild type plants. The higher transcript expression of *APX, CAT, SOD, DREB*, and *AP2* genes were observed in transgenic lines under stress conditions compared to WT and VC plants. The results further support that the *SbSRP* gene could be used as a potential candidate gene to improve salt and osmotic stress tolerance of crop plants. It is also speculated that *Sb*SRP may function as a transporter protein. However, a detailed study is needed to confirm the exact role of the *SbSRP* gene in the abiotic stress tolerance mechanism.

## Author contributions

Conceived and designed the experiments: AM and BJ. Performed the experiments: PU and RJ. Analyzed the data and Wrote the paper: PU and AM.

### Conflict of interest statement

The authors declare that the research was conducted in the absence of any commercial or financial relationships that could be construed as a potential conflict of interest.
