# Supplementary material for: Overexpression of a Plasma Membrane-Localized SbSRP-Like Protein Enhances Salinity and Osmotic Stress Tolerance in Transgenic Tobacco
Source: Front Plant Sci. 2017 Apr 20;8:582. doi: 10.3389/fpls.2017.00582 (PMC5397517; doi:10.3389/fpls.2017.00582)
Supplement: Supplementary file 1 [file DataSheet1.PDF]

Table S1: Primers used in the study.

Primers	Purpose	Sequence (5' to 3')
C-64 R1	5' RACE of *SbSRP* gene	CAATAGGCACAAGAGGCA
C-64 R2		AACAGCTACGTCGGCCAC
C-64 R3		CGTTCTTTGCGATTCC
C-64 F1	3' RACE of *SbSRP* gene	GTGGCCGACGTAGCTGTT
C-64 F2		TATGCCTCTTGTGCCTATTG
SRP-F	Full length, genome organization and Southern blotting	ATGGCAGAAGTTGATT
SRP-R		TCAGTGTGCAGCAGC
Loc-F	Localization	CACCATGGCAGAAGTTGATT
SRPR		TCAGTGTGCAGCAGC
RT-F	Transcript analysis of *SbSRP* gene	AGCGTGTCGGTTGTAGTGGA
RT-R		TCAATAGGCACAAGAGGCA
NtAPX-F	Quantitative real time (qRT) PCR	CAAATGTAAGAGGAAACTCAGAGGA
NtAPX-R		CAGCCTTGAGCCTCATGGTACCG
NtCAT-F		AGGTACCGCTCATTCACACC
NtCAT-R		AAGCAAGCTTTTGACCCAGA
NtSOD-F		AGCTACATGACGCCATTTCC
NtSOD-R		CCCTGTAAAGCAGCACCTTC
NtAP2-F		AAGGGCGAGGAAGAACAAAT
NtAP2-R		GTGGCTCTGGAA AGTTGA
NtDREB-F		GCCGACGCTAAGGATA TTCA
NtDREB-R		TGCAAAACAGAGCTTCCTCA

***SbSRP* gene**

up-stream region (5′UTR)

CCACACGGCTTCACGCAATCACACGGTCTCACTCATTTTTTCTATAAATATTTATATACTCTTCATGAATCATGGCATCATTTCATACTCATTCGTCA
TCAAATTCTCTGTGTTTTCTCTTACTTCCTCTTTTTCTCTCTAGATTTGGAAAGAATCTCGTTTTTTGTTGAATTTGATTCGATTTGGCCAGCGAATT
CATCACCAATCAAATTTATTTCGCGATCTTGATTCTTGTACTTGCTTAACCCTAAGTATTCGCTTTTGATTTTTGTCTGAGGTCTTGATCATTTTAAT

ORF (open reading frame)

ATGGCAGAAGTTGATTCTAAAGTTGATAAACAAACGAGTGGCAATGAAGAACAAAACTTGAAGTACTTTCAGTTTGTACAATCTGCTACATTTCAAGC
TATTGTCTGCTTTTCAAACCTTTATGGTTATGCTAAGGACAAGGCTGGACCCCTGAAGCCTGGTGTTGAAACTGTCGAGGGTACAGTCAAGACTGTTG
TTCGTCCTGTTTATGATCGGTTTCATGGCGTTCCTCTTGAACTCCTTACCTTTGTTGATCGAAAGTTTGATGACACAGTCACAAAGTTTCAGCATCAT
GTTCCCCCCCTAATGAAGCAGATGTCTTTCCAAGCATACTCTGCTGCCCAAAAGACCCCTGAGGTGGCTCGTGGTGTAGCTACAGAAGTTAAGCGTGT
CGGTGTAGTGGATACCGCATCTGGAATCGCAAAGAACGTCTACGTCAAATACGAGCCCACCGCTAAGGAGCTGTATTCCAAGTACGAGCCCGTGGCAG
AGCACTATGCCGTTTCAGCATGGCGATCACTGAATCGTCTTCCCCTTTTCCCAAGAGTGGCCGACGTAGCTGTTCCTGCAGCAGCCCTTTGCACTGAA
AAGTATAACCAAACTGTGAACTTAGGTACTGAAAAGGGTTACAGAATAGCTAATTATATGCCTCTTGTGCCTATTGAGAAGATAGCTAAGGTGTTTAG
GGATGGTGAGACACAGCCTGATGCTGCTAATGGAGACAGTGCTGCTATGCCTGCTGCTGCACACTGA

down-stream region (3′UTR)

GCTTTTGTTGCTTTCTTGTAGAACTTGCAAAACATGGGGATGTTCATAGTTTGAAACTCTTGGTTAAGTAGTAATGCAGTTTTGGTGTTTAGGATGTA
TAAGTTTTTGCTCTGTTTGTATGATTTCATGTTTAAGTTCGGGGACCTTATTTCTTTATTCTTCAGAACTTTTTAGTTCATGATAAATGACATGTAAA
AAGTTGCCCTTTTATCCCGAAAAAAAAAAAAAAAAAAA

poly *A* tail

***Sb*SRP protein**

MAEVDSKVDKQTSGNEEQNLKYFQFVQSATFQAIVCFSNLYGYAKDKAGPLKPGVETVEGTVKTVVRPVYDRFHGVPLELLTFVDRKFDDTVTKFQHH
VPPLMKQMSFQAYSAAQKTPEVARGVATEVKRVGVVDTASGIAKNVYVKYEPTAKELYSKYEPVAEHYAVSAWRSLNRLPLFPRVADVAVPAAALCTE
KYNQTVNLGTEKGYRIANYMPLVPIEKIAKVFRDGETQPDAANGDSAAMPAAAH

Figure S1: Graphical representation of *SbSRP* gene. The *SbSRP* gene was 1262 bp long and consisted of a 5′-untranslated leader sequence (5′-UTR; 1–294 bp), an open reading frame (ORF; 295–1047 bp), a 3′-UTR (1048–1262 bp) and a poly(A) tail of 19 base pairs. The 753-bp ORF encodes a peptide of 250 amino acids.

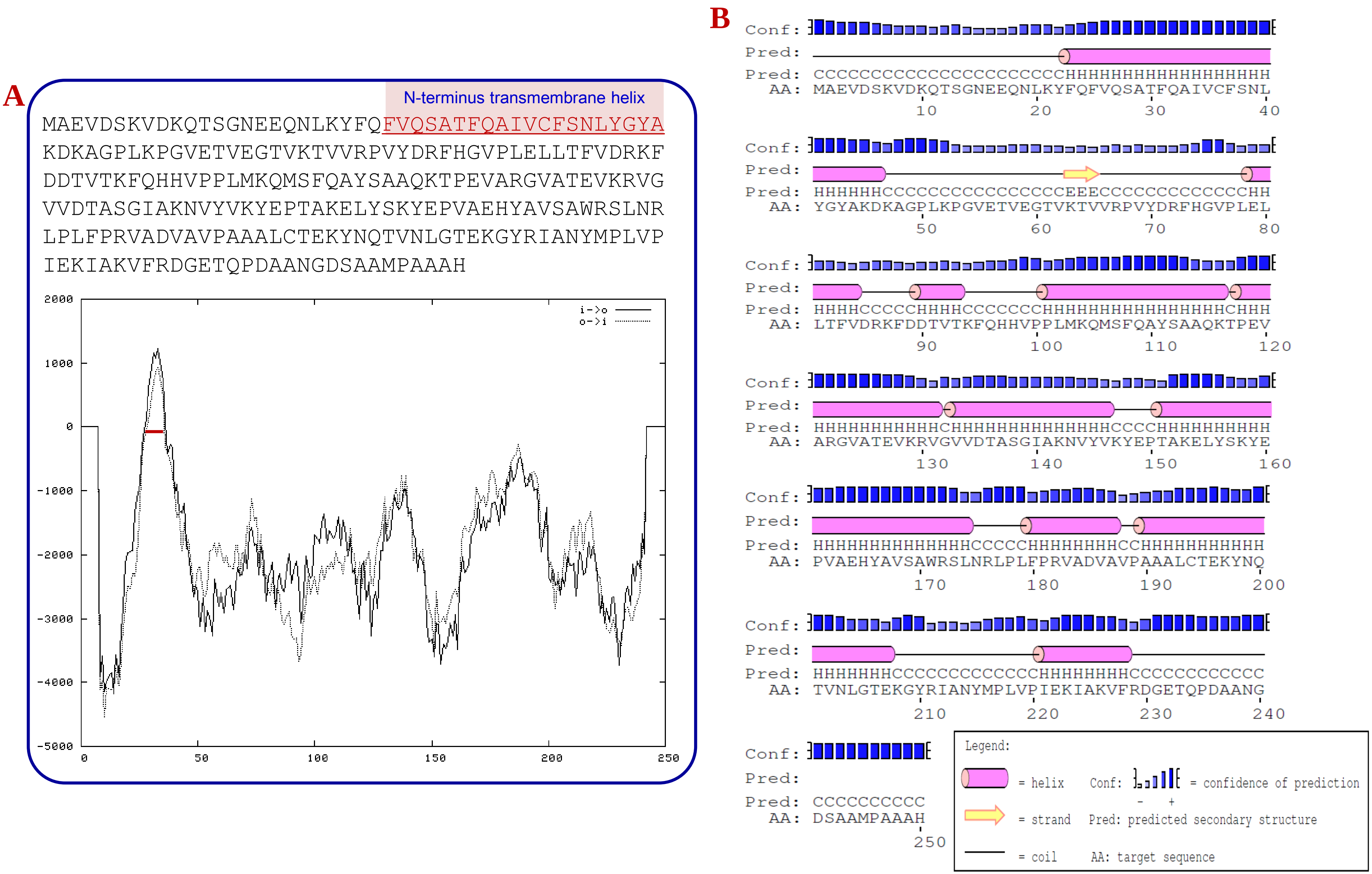

Figure S2: *In silico* analysis of *Sb*SRP. (A) Prediction of transmembrane domain using *TMpred* Program. (B) Secondary structure prediction using PSIPRED.

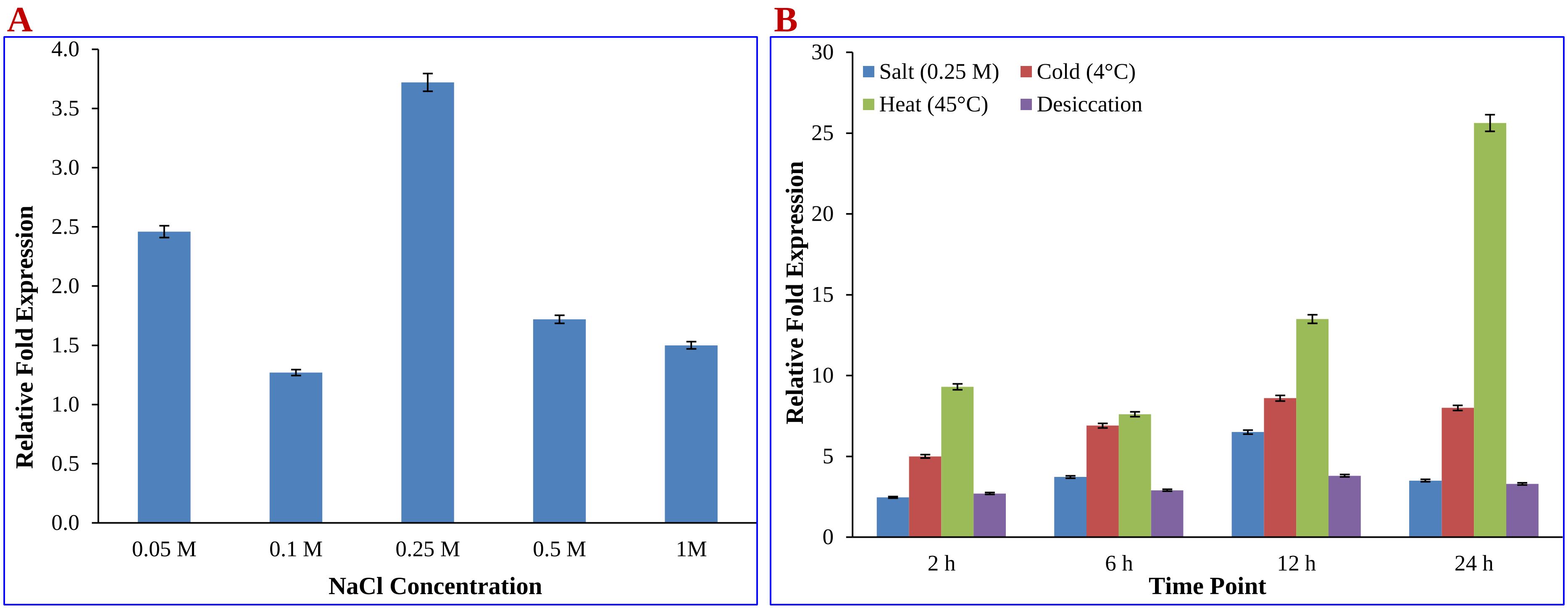

Figure S3: Transcript profiling of the *SbSRP* gene under different abiotic stress condition.

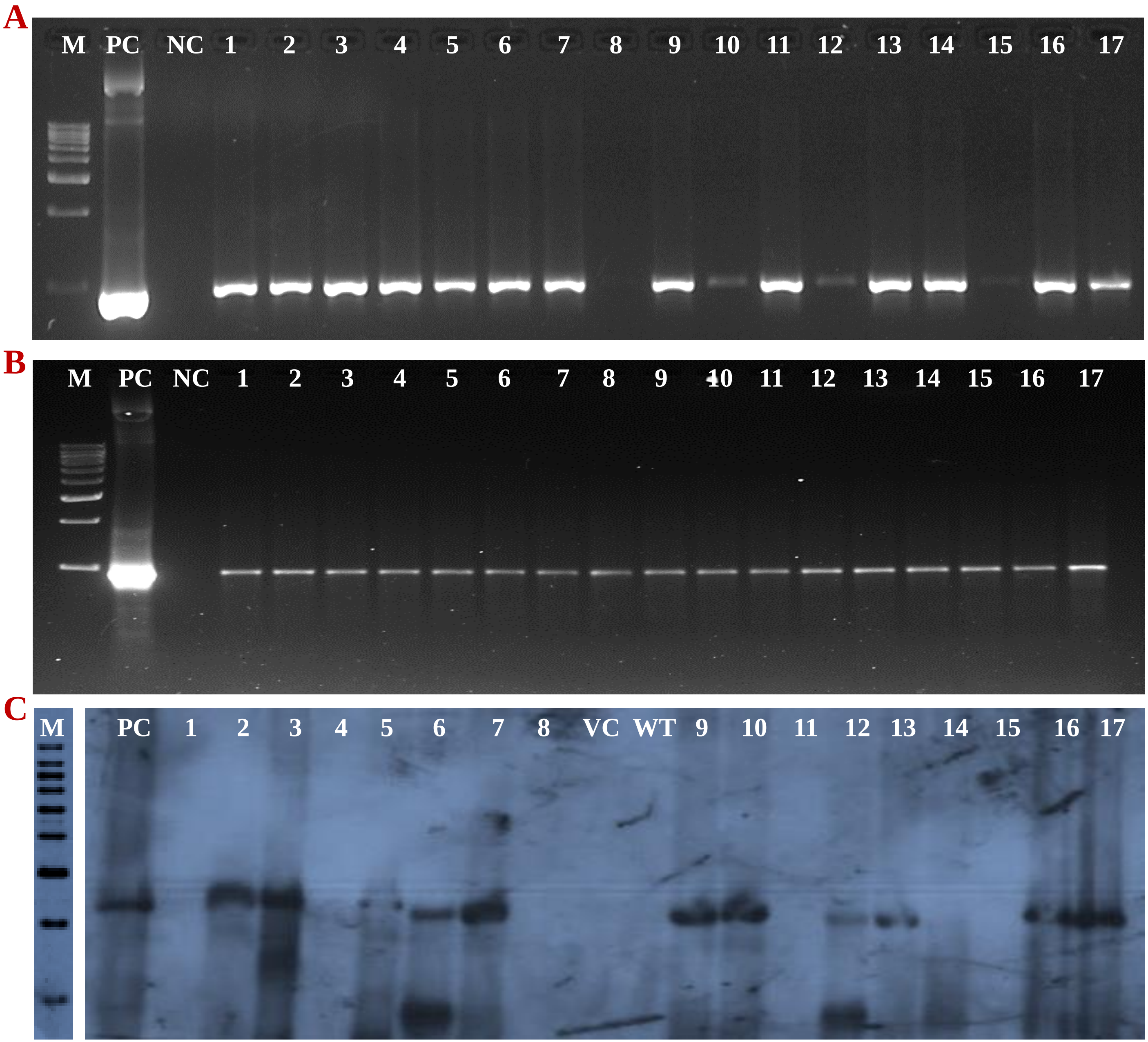

Figure S4: Molecular confirmation of transgenic tobacco plants. PCR amplification of **(A)** *uidA* (gus) and **(B)** *hptII* gene in T1 transgenic lines, WT and VC plants; **(C)** Southern hybridization to determine transgene copy number in transgenic lines. M: molecular marker, PC: positive control, NC: negative control, WT: wild type plant (non-transformed), VC: vector control (transgenic plant transformed with pCAMBIA1301 vector only) and L1-L17: transgenic tobacco lines.

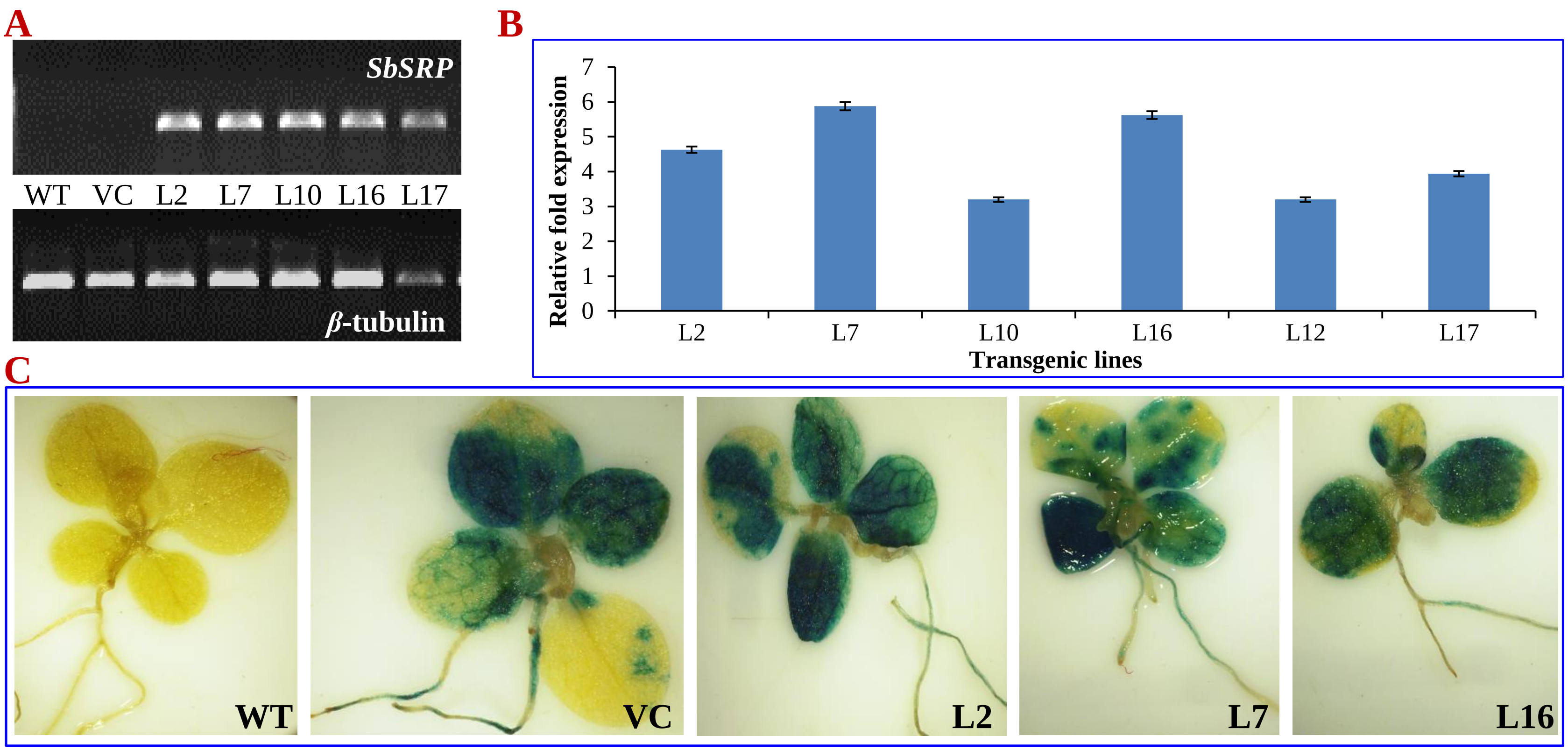

Figure S5: Confirmation of gene expression. (A) Semi-quantitative Rt PCR, **(B)** quantitative real-time (qRT) PCR and **(C)** histochemical GUS assay of selected transgenic lines.

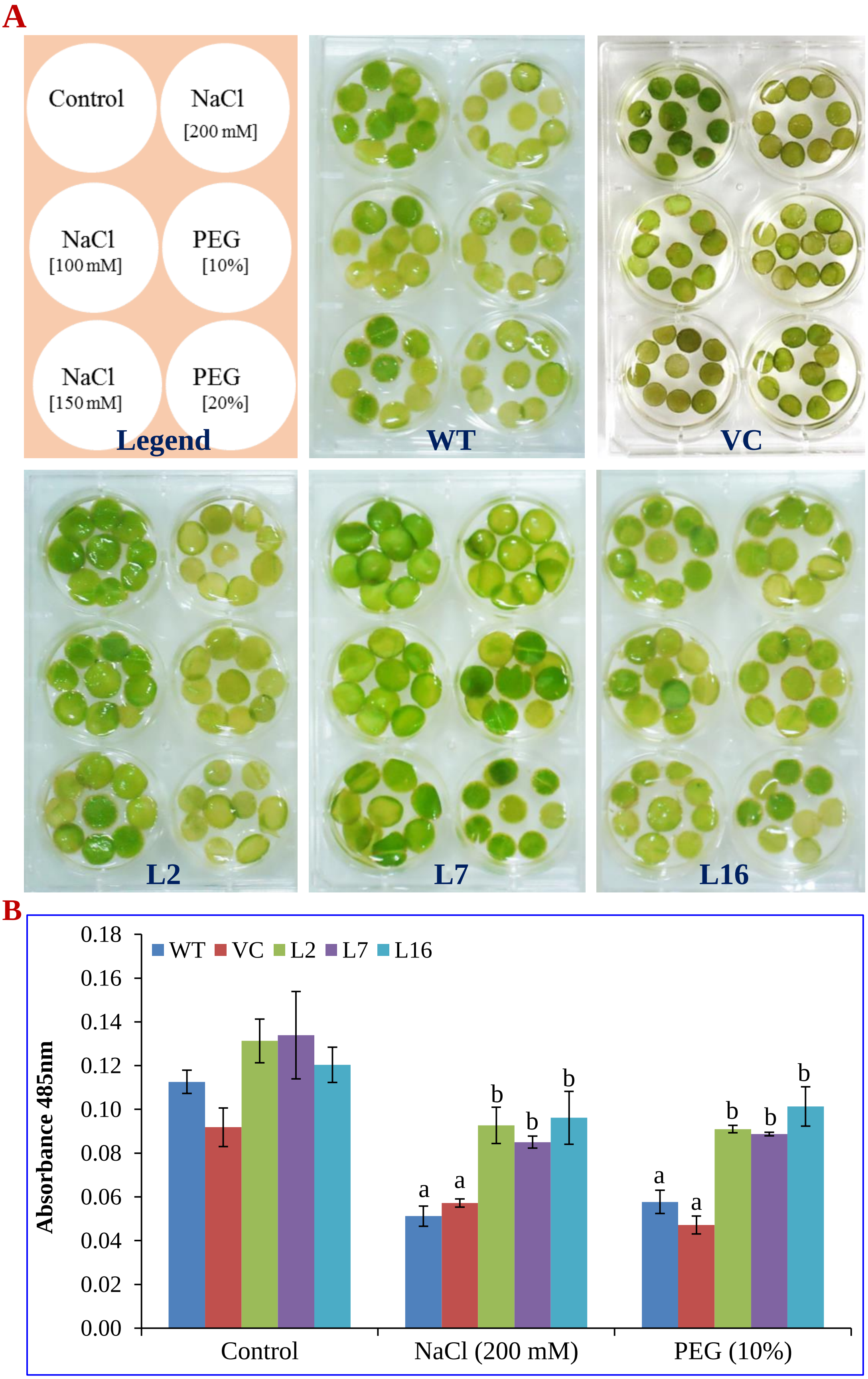

Figure S6: Leaf disc assay and cell viability. (A) Leaf disc assay and **(B)** cell viability of control (WT and VC) and transgenic plants (L2, L7 and L16) for salt and osmotic tolerance under salt and osmotic stress condition. Bars represent means ± SE and values with different letter are significant at *P*<0.05.

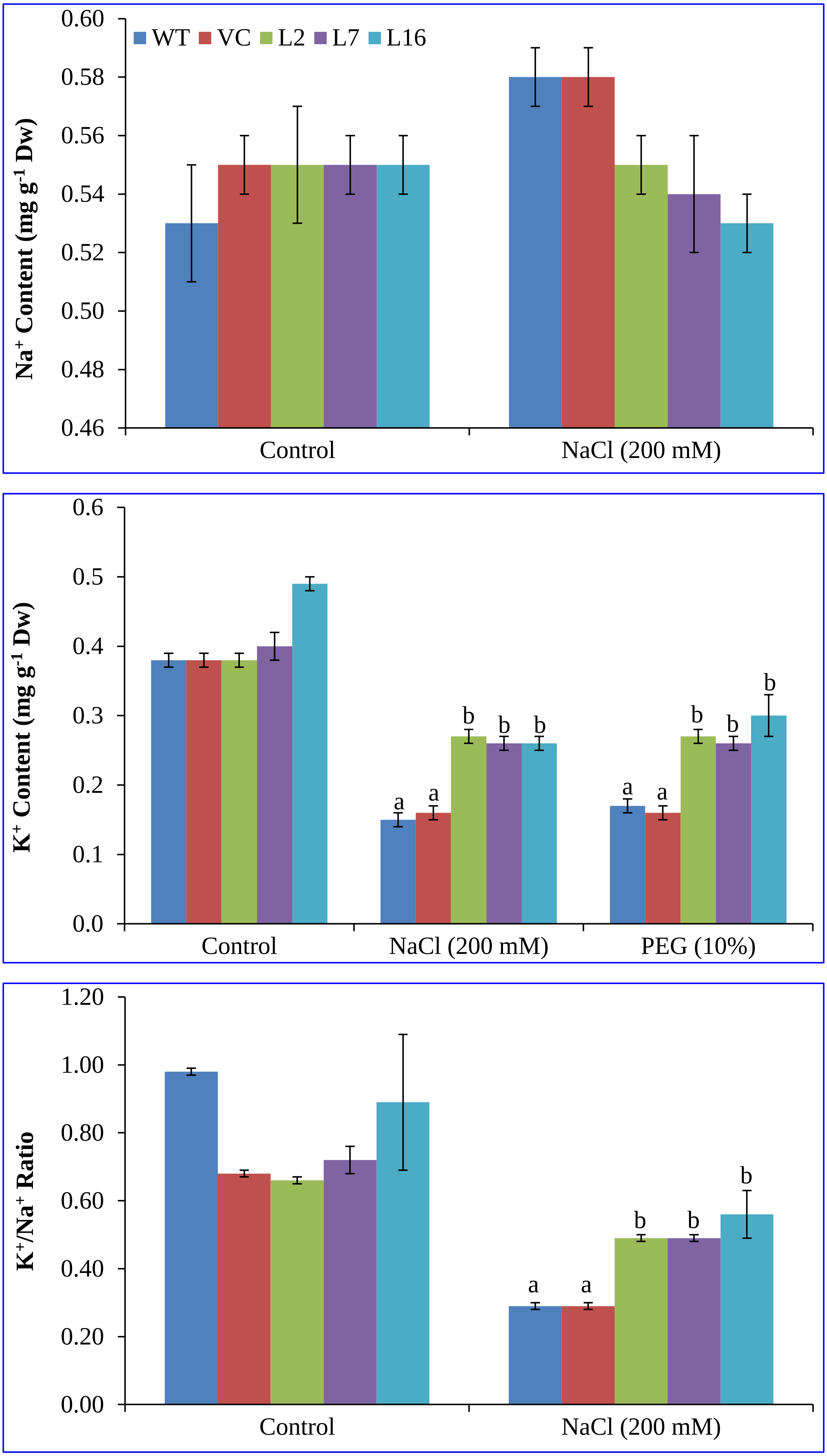

Figure S7: ICP analysis showing Na^+ and K^+ and K^+/Na^+ homeostasis in transgenic lines. Bars represent means ± SE and values with different letter are significant at $P<0.05$.
